# Application of Mesenchymal Stromal Cells and Their Exosomes in Neurodegenerative Diseases and Lysosomal Storage Diseases

**DOI:** 10.3390/cells15171540

**Published:** 2026-08-26

**Authors:** Aisylu I. Ayupova, Angelina S. Sidorova, Ekaterina A. Luzina, Albert A. Sufianov, Galina Z. Sufianova, Azat M. Zaynutdinov, Albert A. Rizvanov, Valeriya V. Solovyeva

**Affiliations:** 1 Institute of Fundamental Medicine and Biology, Kazan Federal University, 420008 Kazan, Russia; ayimullagulova@kpfu.ru (A.I.A.); angssidorova@kpfu.ru (A.S.S.); ekaluzina@kpfu.ru (E.A.L.); vavsoloveva@kpfu.ru (V.V.S.); 2 Department of Neurosurgery, Sechenov First Moscow State Medical University of the Ministry of Health of the Russian Federation (Sechenov University), 119991 Moscow, Russia; sufianov_a_a@staff.sechenov.ru; 3 The Research and Educational Institute of Neurosurgery, Peoples’ Friendship University of Russia, 117198 Moscow, Russia; 4 Department of Pharmacology, Tyumen State Medical University, 625023 Tyumen, Russia; sufarm@mail.ru; 5 Medical and Sanitary Unit of the Kazan Federal University, 420043 Kazan, Russia; azmzajnutdinov@kpfu.ru; 6 Division of Medical and Biological Sciences, Academy of Sciences of the Republic of Tatarstan, 420111 Kazan, Russia

**Keywords:** mesenchymal stromal cells, extracellular vesicles, exosomes, neurodegenerative diseases, lysosomal storage disorders, cell therapy, immunomodulation, neuroinflammation, cross-correction, tissue factor, hemocompatibility

## Abstract

Mesenchymal stromal cells (MSCs) have emerged as a promising therapeutic platform for central nervous system disorders, including neurodegenerative diseases and lysosomal storage disorders (LSDs). This review examines MSC mechanisms of action—paracrine activity, immunomodulation, antioxidant effects, TFEB-mediated autophagy regulation, and enzymatic cross-correction in LSDs—while critically assessing translational challenges. We provide a comparative analysis of MSC sources, administration routes, dosing regimens, and safety profiles, with emphasis on hemocompatibility and thrombotic risks. The evidence base for MSC efficacy in amyotrophic lateral sclerosis, Alzheimer’s disease, Parkinson’s disease, multiple sclerosis, and LSDs is systematically reviewed, highlighting both promising signals and limitations. MSC-derived extracellular vesicles are discussed as a cell-free alternative with improved safety and potential blood–brain barrier interaction. We propose an individualized monitoring framework integrating clinical scales, biomarkers, and neuroimaging. Despite preclinical promise, the field faces major hurdles: product standardization, optimal dosing, and the need for large, randomized controlled trials. The most rational path forward lies in combination strategies—MSCs as adjuncts to gene or enzyme replacement therapy—and engineered platforms for sustained delivery. This review provides a roadmap for translational decision-making and identifies critical gaps that must be addressed before MSC-based therapies can be integrated into routine neurological practice.

## 1. Introduction

Mesenchymal stromal cells (MSCs) are multipotent stromal cells characterized by self-renewal, differentiation capacity, and pronounced immunomodulatory activity. In recent years, MSCs have emerged as one of the most actively studied classes of cell products for immunomodulation and tissue homeostasis support, including in central nervous system (CNS) pathology [1]. Neurodegenerative diseases and lysosomal storage disorders (LSDs) share chronic neuroinflammation, oxidative stress, autophagy dysfunction, and progressive neuronal loss, making them potential targets for cell therapy [2].

The therapeutic potential of MSCs in the CNS is primarily attributed to their paracrine activity: secretion of cytokines, chemokines, growth factors, and extracellular vesicles, as well as contact-dependent interactions with immune cells [3]. MSCs are capable of polarizing microglia from a pro-inflammatory (M1) to a reparative (M2) phenotype, reducing oxidative stress via activation of the nuclear factor erythroid 2-related factor 2 (Nrf2) signaling pathway, inhibiting neuronal apoptosis through modulation of Bcl-2/Bax and caspase expression, and stimulating autophagy and the transcription factor EB (TFEB) signaling pathway [3,4]. Moreover, for LSDs, the ability of MSCs to mediate enzyme cross-correction, the secretion of lysosomal hydrolases with subsequent uptake by deficient cells, is of particular importance [5].

Key determinants for clinical translation include the MSC source, route of administration, dosing regimen, treatment course frequency, and safety profile. MSC-derived extracellular vesicles (MSC-EVs) are emerging as an alternative strategy, as they retain therapeutic mechanisms while offering a better safety profile and the potential to cross or interact with the blood–brain barrier (BBB) [6].

This article provides a comprehensive overview of MSC mechanisms of action, a comparative characterization of cell sources, administration routes, treatment regimens, and the evidence base for neurodegenerative diseases and LSDs and also discusses monitoring, logistics, and prospects for combination therapy.

What distinguishes this review is its side-by-side comparison of multiple key variables that are usually discussed separately: MSC tissue sources, routes of administration, dosing schedules and treatment frequency, hemocompatibility and tissue factor-mediated risks, as well as the specific role of cross-correction in LSDs. In parallel, we provide a critical appraisal of MSC-derived extracellular vesicles as a cell-free platform, highlighting both their promise and the current technological bottlenecks. By integrating these dimensions within a single framework, we aim to offer a practical roadmap for translational decision-making and to identify gaps that need to be addressed before MSC-based therapies can move confidently into routine neurological practice.

The literature search for this review was conducted across multiple electronic databases, including PubMed/MEDLINE, Web of Science, Scopus, and Google Scholar, supplemented by searches of clinical trial registries (ClinicalTrials.gov and WHO ICTRP) without date restrictions to capture publications from 2000 to 2026. The search strategy employed combinations of keywords and terms related to mesenchymal stromal cells, extracellular vesicles/exosomes, neurodegenerative diseases, lysosomal storage disorders, mechanisms of action (immunomodulation, paracrine activity, TFEB signaling, cross-correction), and translational aspects (administration routes, dosing, safety, hemocompatibility, clinical trials). Studies were included if they addressed MSC biology, therapeutic applications in CNS disorders, MSC-derived EVs, or clinical investigations, with priority given to English-language publications presenting original data or comprehensive synthesis. The selection process involved initial screening of titles and abstracts followed by full-text review, reference mining, and quality assessment, ultimately yielding approximately 800 studies for qualitative synthesis (including preclinical studies, clinical trials, review articles, and case reports). Evidence was classified according to a hierarchical system (Class I–IV) to differentiate between high-level clinical trial data, observational studies, and preclinical findings, with explicit indication of evidence level in the manuscript to provide readers with a clear understanding of the strength and limitations of the presented data.

## 2. Mechanisms of MSC Action

### 2.1. Paracrine Activity and MSC Secretome

MSCs are multipotent stromal cells characterized by self-renewal and differentiation capacity, as well as pronounced immunomodulatory properties. MSCs are considered one of the most actively studied classes of cell products for immunomodulation and tissue homeostasis support [1]. The primary therapeutic potential of MSCs is attributed to their paracrine activity, which includes the secretion of cytokines, chemokines, growth factors, and extracellular vesicles (EVs), as well as contact-dependent interactions with effector cells of the immune system [1,7,8]. After administration, MSCs rapidly respond to signals of injury and inflammation in the microenvironment, including within the CNS. In response to these stimuli, the cells secrete biologically active molecules that modulate the functional activity of immune cells, promote neuronal survival, and stimulate neurite outgrowth. MSCs have been shown to secrete a broad spectrum of growth factors and cytokines that play a key role in mediating neuroprotective effects [3]. These include neurotrophic and angiogenic factors such as vascular endothelial growth factor (VEGF), basic fibroblast growth factor (bFGF), hepatocyte growth factor (HGF), insulin-like growth factor-1 (IGF-1), brain-derived neurotrophic factor (BDNF), nerve growth factor (NGF), glial cell line-derived neurotrophic factor (GDNF), as well as anti-inflammatory mediators such as transforming growth factor-β (TGF-β), prostaglandin E2 (PGE2), and interleukin (IL)-10, which underpin their anti-apoptotic, pro-angiogenic, immunomodulatory, and neuroprotective potential [9,10]. The quantitative ratio of these molecules depends on the MSC source and culture conditions [11]. In addition to secreting soluble protein factors, MSCs produce EVs enriched with microRNAs (miRs) (in particular, miR-21, miR-23, miR-124, miR-130, and others). Through the transfer of these regulatory molecules, EVs modulate the expression of genes associated with neuronal survival, angiogenesis, and the inflammatory response. This mechanism is supported by findings from functional studies demonstrating the effects of purified MSC-derived vesicles on target neuronal and endothelial cells [7,12]. Furthermore, in vitro experiments have shown that conditioned medium obtained from MSC cultures promotes increased neuronal survival under stress conditions and stimulates neuritogenesis [13].

### 2.2. Immunomodulatory Mechanisms of MSCs in the CNS

The therapeutic potential of MSCs in CNS diseases whose pathogenesis is associated with neuroinflammation and peripheral immune activation is attributed to their immunomodulatory properties [1]. A key mechanism of MSC action is the reprogramming of microglia and infiltrating immune cells toward a neuroprotective phenotype. Microglia, as central mediators of neuroinflammation, can polarize from a pro-inflammatory (M1) to a reparative (M2) phenotype, which differ in their cytokine profiles and effects on neuronal survival [14].

**Figure 1:**
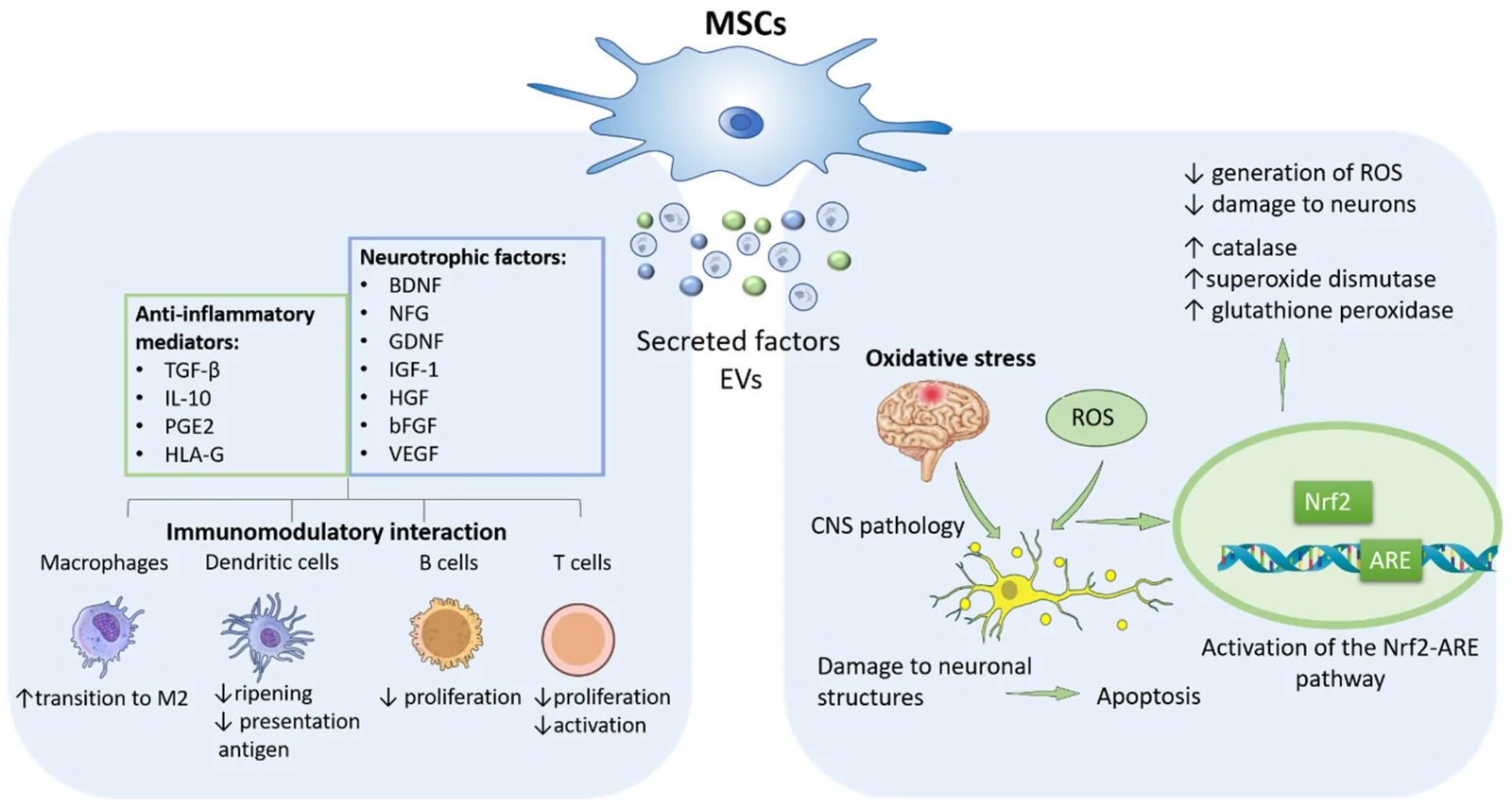
Immunomodulatory and antioxidant effects of MSCs.

In a model of Niemann–Pick disease (NPD) type C, administration of bone marrow-derived MSCs (BM-MSCs) reduced astrocyte and microglial activation, correlating with enhanced neuroprotection [4]. In a mouse model of traumatic brain injury, intravenous (IV) administration of BM-MSCs induced expression of M2 phenotype markers in microglia and macrophages on days 3 and 7, accompanied by a sustained reduction in CD68 expression and improved neurological outcomes compared to controls [15]. In a rat model of focal cerebral ischemia, MSC transplantation promoted the switch of the microglia/macrophage profile from M1 to M2, leading to reduced levels of pro-inflammatory mediators and improved functional recovery (Figure 1) [16].

The immunoregulatory activity of MSCs is modulated by signals from the inflammatory microenvironment. In particular, stimulation with pro-inflammatory cytokines (interferon (IFN)-γ, tumor necrosis factor (TNF)-α) enhances the immunosuppressive potential of MSCs [17]. MSCs suppress the production of pro-inflammatory cytokines and modulate the activity of a wide range of immune cells, including T- and B-lymphocytes, NK cells, monocytes/macrophages, and dendritic cells. With respect to innate immunity, a key mechanism is the reprogramming of macrophages toward an anti-inflammatory phenotype [18]. With respect to adaptive immunity, MSCs exert a dual effect: on one hand, they suppress T-lymphocyte proliferation and their production of pro-inflammatory cytokines (IFN-γ, TNF-α); on the other, they promote the differentiation and functional activation of regulatory T-cells (Tregs). The latter process is achieved both directly and indirectly—through modulation of antigen-presenting cells [19,20]. In the context of neuroinflammation, the therapeutic effect of MSCs can be reproduced using their EVs. In an acute spinal cord injury model, administration of EVs derived from human MSCs was shown to reduce the severity of neuroinflammation and promote functional recovery [21]. Furthermore, the clinical relevance of the immunomodulatory properties of MSCs has been confirmed in a Phase II study in patients with relapsing-remitting multiple sclerosis (MS). Administration of autologous BM-MSCs was associated with a reduction in the cumulative number of gadolinium-enhancing lesions compared to placebo, which is considered a clinically significant anti-inflammatory effect [22]. Thus, immunomodulation is a key mechanism through which MSCs attenuate neurodegenerative processes associated with chronic neuroinflammation.

### 2.3. Antioxidant Effects and Inhibition of Apoptosis

Neurodegenerative processes are inextricably linked to oxidative stress—the accumulation of reactive oxygen species (ROS) that induces damage to neuronal structures and triggers programmed cell death, which act as interconnected components of the pathogenesis of several CNS disorders, including ischemic stroke, where ROS initiate pro-apoptotic signaling cascades [23]. MSCs and their secretome are capable of reducing oxidative stress levels by activating endogenous antioxidant systems in target cells [24,25]. The antioxidant potential of MSC-derived EVs has been demonstrated by Luo et al. EVs enriched with antioxidant microRNAs increased the activity of catalase, superoxide dismutase, and glutathione peroxidase and reduced ROS generation and the level of oxidative damage to biomolecules in hippocampal neurons under oxidative stress in vitro and in a seizure injury model in vivo [26]. The critical role of the Nrf2 signaling pathway was confirmed by experiments with its blockade, which abrogated the protective effect of the EVs [26]. Similar results were obtained for exosomes derived from adipose tissue MSCs (AD-MSCs) in a model of methotrexate-induced neurotoxicity, where protection was also mediated by activation of the Nrf2-antioxidant response element (ARE) pathway [27].

Under co-culture conditions, MSCs reduce neuronal apoptosis induced by β-amyloid and other pathogenic factors in Alzheimer’s disease (AD) and Parkinson’s disease (PD). In a PD model, MSC transplantation increased the survival of dopaminergic neurons and the expression of tyrosine hydroxylase [4]. The anti-apoptotic effect of MSCs has been confirmed in models of ischemic injury: under oxygen–glucose deprivation of mixed neuronal-glial cultures, co-culture with MSCs reduced the number of TUNEL-positive cells (TUNEL—terminal deoxynucleotidyl transferase dUTP nick end labeling) and *Bax* expression. Blockade of *Bcl*-2 completely abolished the protective effect, indicating a key role for activation of the anti-apoptotic *Bcl*-2 pathway [28]. In a spinal cord injury model, MSC-derived exosomes induced an increase in the Bcl-2/Bax ratio and reduced the levels of caspases-3 and -9 [29]. Collectively, the presented data indicate that MSCs and their EVs effectively reduce oxidative stress and suppress neuronal apoptosis in the CNS.

### 2.4. Modulation of Autophagy and TFEB Signaling

Autophagy and the autophagolysosomal pathway (ALP) ensure the clearance of damaged proteins and organelles in neurons and glia; ALP dysfunction is associated with the progression of neurodegeneration [3]. A key regulator of autophagy and lysosomal biogenesis is TFEB. Under conditions of mammalian target of rapamycin (mTOR)-dependent phosphorylation, TFEB is retained in the cytoplasm [30]. In the context of neurodegeneration, enhancement of TFEB-mediated clearance can reduce the toxicity of aggregating proteins and slow disease progression [31].

MSCs can stimulate autophagy in affected neurons through TFEB activation. In a model of spinocerebellar ataxia type 3 (SCA3), co-culture with MSCs induced nuclear translocation of TFEB, restored autophagy, reduced the number of toxic protein aggregates, and increased neuronal survival [3]. These findings establish a direct causal link between MSC-mediated TFEB activation and therapeutic outcomes in SCA3 models.

By contrast, for ischemic injury, similar mechanisms have been described only as a working hypothesis. MSC-derived exosomes have been shown to activate autophagy via miRNAs (e.g., miR-421), which is potentially coupled to TFEB-dependent pathways, and reduce apoptosis and neuroinflammation in models of oxygen–glucose deprivation and transient middle cerebral artery occlusion [32,33]. However, in these ischemia/miRNA models, the direct involvement of TFEB—rather than parallel autophagy-regulatory routes—remains inferred from correlative data and has not been verified through TFEB-specific loss- or gain-of-function experiments within the MSC/exosome setting.

Importantly, TFEB signaling in microglia is involved in the control of inflammatory cascades. TFEB has been shown to negatively regulate the NLRP3 inflammasome via lysosomal-associated membrane protein (LAMP)-2A-dependent chaperone-mediated autophagy, whereas TFEB suppression promotes NLRP3 activation and neuroinflammation in a PD model [34]. Taken together, the assertion that MSCs modulate autophagy in the CNS primarily through TFEB activation—leading to enhanced lysosomal degradation, reduced neuroinflammation, and increased neuronal survival—is currently supported as a unifying concept primarily in selected models (notably SCA3), but for the majority of diseases and injury paradigms, the direct in vivo contribution of TFEB to MSC therapy remains hypothetical and requires rigorous confirmation through pathway-specific intervention studies.

### 2.5. Plasticity and Differentiation Potential of MSCs

Although paracrine activity is considered the primary mechanism of MSC action, the literature indicates their phenotypic plasticity. A proportion of transplanted MSCs has been shown to migrate to the site of CNS injury and acquire features of cells resembling astrocytes, glia, and neurons. Neural differentiation of MSCs in vitro has been confirmed by numerous studies demonstrating the induction of neuron-like morphology and expression of neural markers (neuronal nuclei (NeuN), neuron-specific enolase (NSE), glial fibrillary acidic protein (GFAP)) under specific culture conditions [35]. However, marker expression alone is not sufficient evidence of functional neuron formation. It has been shown that undifferentiated MSCs can constitutively express a number of neural markers, indicating a lack of strict specificity of the latter [36]. A more reliable criterion for neuronal differentiation is the presence of functional signs of maturity: electrophysiological activity, synaptic transmission, and neuronal network formation [37].

With respect to in vivo cell replacement in the CNS, the evidentiary requirements are substantially higher. Necessary conditions include confirmation of transplanted cell survival, migration, acquisition of a neural/glial phenotype in the tissue, and evidence of interaction with the host microenvironment. According to an analysis of preclinical data, neural differentiation is likely not a dominant property of adult MSCs in the context of therapy for neurological pathologies; even when individual observations of neuron-like features exist, the primary therapeutic effect is not reduced to direct neuronal replacement [38]. For instance, in a Krabbe disease model, xenotransplanted MSCs were detected in the mouse brain with expression of neuronal, astrocytic, and oligodendrocytic markers, but their engraftment and sustained presence in the tissue remained limited, and conclusive evidence of functional participation in neural circuits has not been established [39].

Cross-correction in LSDs represents a distinct, enzyme-based mechanism that has been extensively investigated in preclinical settings. The concept is that a functionally active lysosomal enzyme synthesized by a healthy cell can be secreted, taken up by enzyme-deficient cells, and transported to lysosomes, partially restoring catabolic function [5,40]. The molecular basis of this process is the presence of a mannose-6-phosphate (M6P) tag on many lysosomal hydrolases, which ensures binding to the cation-independent M6P receptor on the surface of target cells, followed by internalization and delivery to the lysosomal compartment. In the CNS, the significance of cross-correction is determined by the pronounced neurological manifestations of enzyme-deficient conditions coupled with the limited availability of exogenous enzymes due to the BBB [41].

For unmodified MSCs, the clinical evidence for cross-correction remains limited to isolated observations. MSCs from healthy donors, possessing normal lysosomal enzyme activity, are theoretically capable of compensating for enzyme deficiency in patients with LSDs. When allogeneic MSCs were administered to patients with metachromatic leukodystrophy, arylsulfatase A levels in the CNS increased, with normalization of activity at 16 months [42]. However, these represent restricted clinical observations rather than systematically validated outcomes, and the direct causal link to MSC-mediated cross-correction in humans requires further substantiation.

In contrast, genetically modified MSCs provide a robust preclinical rationale for enhancing cross-correction. In Tay–Sachs disease, unmodified MSCs secrete insufficient amounts of β-hexosaminidase A (HexA), which justifies the strategy of genetic modification. MSCs transfected with the *HEXA*/*HEXB* genes produce active enzymes and provide cross-correction of the deficiency in patient cells in vitro; upon intravenous administration to rats, HexA is detected in plasma, brain, and spinal cord without signs of immune rejection [5]. Importantly, these findings remain at the preclinical stage, and the translational feasibility of this approach—including long-term enzyme expression, biodistribution, and safety—has yet to be established in clinical settings.

Thus, MSCs represent a multifunctional modulator of the CNS microenvironment, whose therapeutic potential is thought to derive less from direct neuronal replacement and more from their multifaceted impact on key pathogenetic processes. Through paracrine secretion of neurotrophins, cytokines, and EVs, MSCs provide neuroprotection, stimulate angiogenesis and reparative plasticity, while simultaneously reprogramming the inflammatory response from a destructive to a regenerative profile. Immunomodulation of microglia, antioxidant defense, regulation of autophagy, and the capacity for cross-correction of enzymatic deficiencies—the latter currently supported primarily by preclinical data and selected clinical observations—complement this spectrum of effects, positioning MSCs as a versatile therapeutic tool for both acute CNS injuries and progressive neurodegenerative diseases.

Although MSC mechanisms are well validated in preclinical models, their clinical relevance remains unclear. These effects may be transient, dose-dependent, or insufficient for chronic neurodegenerative pathology, and their synergy or redundancy in patients is unproven. Preclinical data do not guarantee efficacy, so clinical trials should include functional monitoring of the cell product—viability, phenotype, secretory activity, and potency assays—alongside clinical endpoints to clarify whether product variability affects patient responses.

## 3. Comparison of MSC Sources: Safety, Potency, Hemocompatibility, and Secretory Profile

MSCs are isolated from a wide range of tissues; however, for data comparability, it is fundamentally important to always specify the tissue of origin, as cells from different sources may differ in phenotype, functional properties, and secretory profile. In the current literature, two broad groups of sources are most commonly distinguished: adult and perinatal tissues [43]. Classically, MSCs were isolated from bone marrow, but other sources are now available, including adipose tissue, umbilical cord blood (UCB-MSC), and umbilical cord tissue (UC-MSC)/Wharton’s jelly (WJ-MSC). MSCs from the placenta, amniotic membrane, dental pulp, and other sources are also being investigated. All of these meet the criteria for MSCs, such as adherence to plastic, the CD73^+^CD90^+^CD105^+^ phenotype, and the capacity for osteogenic, chondrogenic, and adipogenic differentiation in vitro. However, functionally, different sources exhibit differences in proliferative activity, immunological properties, and secretory profiles [4,44].

### 3.1. Bone Marrow-Derived MSCs

Bone marrow is the most extensively studied and classic source of BM-MSCs. BM-MSCs are obtained from bone marrow aspirate followed by isolation of the adherent fraction and culture expansion; the properties of the final cell product depend substantially on culture conditions and the donor’s baseline characteristics [45]. Disadvantages of this source include the invasiveness of the harvesting procedure, limited cell yield from the starting material, and the need for 2–3 weeks of culture to achieve a therapeutic dose. Furthermore, BM-MSCs demonstrate lower secretion of neurotrophic factors. Nevertheless, the immunosuppressive activity of BM-MSCs remains the gold standard, and considerable clinical experience has been accumulated with their use [13].

A critical parameter upon intravascular administration is hemocompatibility: contact of the cell product with blood can trigger the instant blood-mediated inflammatory reaction (IBMIR), associated with a risk of thrombotic complications and reduced survival of transplanted cells [46]. BM-MSCs are often considered more hemocompatible for intravenous administration compared to alternative sources, as they are characterized by relatively low expression of tissue factor (TF/CD142)—a key trigger of the coagulation cascade [20,46]. However, it is essential to emphasize that hemocompatibility is not determined by tissue source alone but rather by the specific cell product characteristics, including TF/CD142 expression levels, the presence of cell aggregates, viability, passage number, culture conditions, washing procedures, dose, and infusion rate. Consequently, while BM-MSCs may offer a favorable profile in comparative studies, product-specific hemocompatibility testing remains mandatory prior to clinical intravenous administration.

BM-MSCs possess pronounced immunomodulatory activity. Comparative studies demonstrate their higher capacity to suppress T-lymphocyte proliferation relative to some other MSC types, which is attributed to elevated expression levels of the immunosuppressive cytokines IL-10 and TGF-β1 [13,47]. At the same time, BM-MSCs are characterized by high osteogenic and chondrogenic differentiation capacity. This underpins their demand in orthopedic indications [44]. Notably, according to comparative studies, differences between tissue sources of MSCs do exist; however, the final functional activity is equally influenced by culture conditions, donor variability, and standardization of testing methods [47,48]. These sources of variability may affect MSC phenotype, secretome composition, and functional potency. In addition, differences in culture media, oxygen conditions, passage number, expansion protocols, and manufacturing procedures may contribute to variability between MSC products and clinical studies. From a clinical safety perspective, BM-MSCs have the largest body of observational data, including use in neurological pathology. In a study of repeated intrathecal administration of allogeneic BM-MSCs to patients with CNS diseases, the injections demonstrated good tolerability, with no serious adverse events (AEs); the most common reactions were transient local pain, low-grade fever, and headache, with no signs of infectious complications or tumor growth reported [49].

### 3.2. Adipose Tissue-Derived MSCs

Adipose tissue represents the most accessible and scalable source of AD-MSCs, obtained from the stromal vascular fraction during liposuction or resection. The harvesting procedure is minimally invasive and allows for the isolation of up to 500,000 viable MSCs per gram of tissue regardless of donor age. This yield is tens of times higher than the yield of BM-MSCs, making AD-MSCs optimal for clinical production of large cell volumes [50,51]. AD-MSCs are characterized by high proliferative potential in vitro and secrete a number of factors (bFGF, IGF-1, IFN-γ) in large quantities; several studies have demonstrated their more pronounced immunomodulatory effect compared to BM-MSCs [44]. According to a comparative analysis, both populations are capable of suppressing immune cell activation and proliferation; however, differences in functional parameters and mediator profiles have been identified [52].

AD-MSCs exhibit procoagulant activity, driven by expression of TF/CD142. Upon contact with blood, cell products can initiate IBMIR, with a strategy of selecting grafts with low TF expression being considered a means of reducing thrombotic risk upon intravascular administration [46,53]. AD-MSCs express higher levels of TF compared to BM-MSCs, which is associated with an increased risk of thrombotic complications. In animal experiments, administration of freshly isolated AD-MSCs led to pulmonary thromboembolism and death in the majority of subjects, whereas TF blockade or removal of factor VII from plasma prevented coagulopathies [20]. Culture senescence of AD-MSCs is accompanied by a reduction in the proportion of TF-positive cells; however, in practice, cultures typically do not reach this limit.

Systematic safety evaluation of AD-MSCs in clinical studies confirms the feasibility of such interventions; however, thromboembolic complications have been observed precisely upon systemic administration of cells, underscoring the need for rigorous hemocompatibility control and the development of appropriate protocols (NCT02052427, NCT01556022, NCT00426868, NCT01502514) [54]. Overall, AD-MSCs are positioned as a practically scalable source with well-characterized immunoregulatory properties; however, their use upon intravascular administration requires careful assessment of TF-mediated procoagulant activity and standardization of cell product quality control.

### 3.3. Umbilical Cord Blood-Derived MSCs

Umbilical cord blood is a perinatal source of UCB-MSCs, the procurement of which does not require invasive procedures for the donor, rendering this approach ethically acceptable [55]. At the same time, UCB-MSCs are characterized by low reproducibility of primary isolation: according to the literature, the success rate of isolation, even in optimized protocols, does not exceed 63%, whereas Wharton’s jelly MSCs can be isolated from virtually 100% of samples [56]. A limitation is the requirement to initiate processing within 8 h after delivery, underscoring the critical dependence of UCB-MSCs on pre-analytical factors. The immunological profile of UCB-MSCs is characterized by minimal expression of human leukocyte antigens (HLA) class I and absence of HLA class II, which reduces the risk of rejection upon allogeneic transplantation. The secretome of UCB-MSCs exhibits the greatest diversity and richness of composition compared to adult-derived MSCs [57]. The paracrine activity of UCB-MSCs is considered a key functional component: comparative studies have demonstrated their anti-inflammatory effect in a lipopolysaccharide (LPS)-stimulated macrophage model involving angiopoietin (Ang)-1 [58].

The clinical safety and efficacy of UCB-MSCs have been confirmed in a study on cerebral palsy, where cell infusion combined with rehabilitation was associated with improved motor and functional outcomes without serious adverse events [59]. Thus, UCB-MSCs represent a perinatal source with high proliferative potential and a favorable immune profile; however, their clinical application is limited by variable isolation success rates and critical dependence on collection and cryopreservation conditions.

### 3.4. Wharton’s Jelly and Umbilical Cord Tissue-Derived MSCs

Perinatal tissues, including the umbilical cord and placenta, are among the most sought-after sources of MSCs due to the combination of non-invasive biomaterial collection and high reproducibility of cell culture isolation. Perinatal MSCs are characterized as biologically young cells with high proliferative potential and low immunogenicity.

The placenta represents a heterogeneous tissue compartment comprising maternal and fetal structures, which accounts for the variability of isolated MSC populations depending on the harvest site and culture protocols [60]. A key advantage of umbilical cord tissue is the high probability of successful WJ-MSC isolation from virtually all samples, including when processing is delayed for up to 48 h. This process is technically simpler compared to UCB-MSC isolation [56].

The secretory profile of UC-MSCs is distinguished by high diversity of biologically active molecules. According to comparative proteomic analysis, fetal MSCs secrete a broader spectrum of proteins, including neurotrophic factors, compared to adult BM/AD-MSCs [61]. WJ-MSCs have been shown to more actively stimulate axonal growth and provide protection to neural stem cells in models of oxidative stress, supporting their promise for neurological applications [13].

A critical safety aspect of UC-MSCs, similar to AD-MSCs, is the high expression of TF, substantially exceeding that of BM-MSCs [20]. It was precisely on UC-MSCs and AD-MSCs that IBMIR was first described, necessitating caution in clinical translation. Risk mitigation strategies include dose reduction upon intravenous administration, thorough washing of cells to remove serum factors and microparticles, as well as selection of lines with low TF expression [62].

Hemocompatibility is of critical importance upon intravenous administration of MSCs and depends on the level of TF expression, which varies according to the source. For systemic infusion, BM-MSCs are preferred, whereas AD-MSCs and UC-MSCs require thorough purification. MSCs do not express co-stimulatory molecules or HLA-DR; this underlies their immune privilege and enables allogeneic use without immunosuppression, particularly with a limited treatment course [4]. Cases of antibody formation upon repeated administrations have been described, including a weakly positive Coombs test; however, no clinically significant rejection reactions have been reported.

Comparative analysis of key characteristics of MSCs from major sources is presented in Table 1.

## 4. Routes of MSC Administration in the Context of CNS Delivery

**Figure 2:**
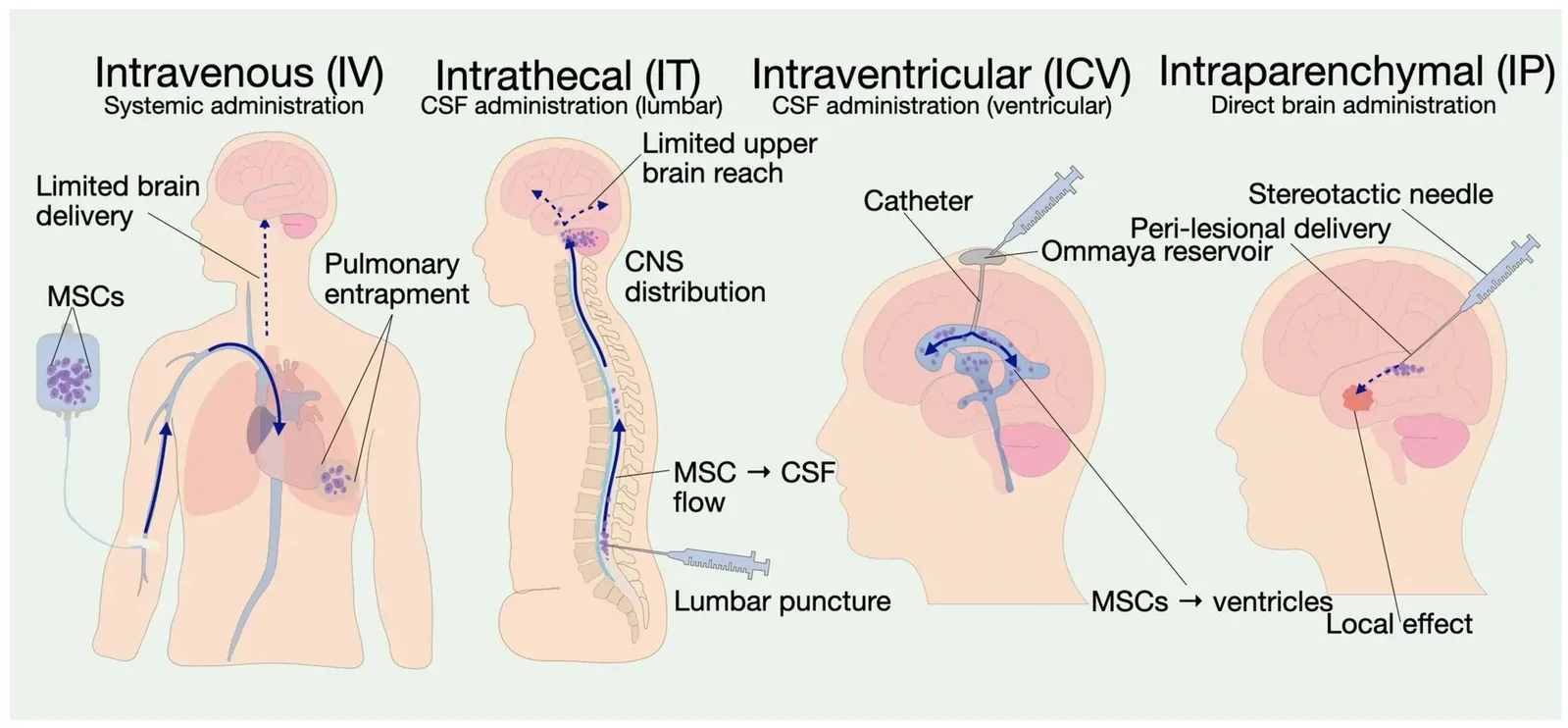
The main routes of delivery of mesenchymal stromal cells to the central nervous system.

The choice of the optimal route for delivering MSCs to the CNS is determined by the need to maintain a balance between the invasiveness of the procedure, the ability to cross the BBB, and the required extent of cell distribution within the brain parenchyma (Figure 2).

### 4.1. Intravenous Administration

The intravenous route remains the most common method of systemic MSC administration due to its technical simplicity and reproducibility. Following infusion, a significant proportion of cells become entrapped in the pulmonary microcirculatory bed, as confirmed by experimental studies [66,67]. The magnitude of this effect is determined by cell size, the presence of microaggregates, infusion rate, and vascular bed characteristics [68]. Pulmonary entrapment fundamentally reduces the likelihood of significant numbers of intact MSCs reaching the brain parenchyma, shifting the focus toward systemic mechanisms of action. Detection of MSCs in CNS tissue after intravenous administration is quantitatively limited [67,69]. In this context, clinical improvements in CNS diseases are more appropriately interpreted as the result of immunomodulatory and tissue-protective cascades initiated by the systemic interaction of the cell product with blood and peripheral immune compartments [70]. In the presence of neuroinflammation or BBB disruption, a small percentage of MSCs may migrate to sites of injury [4].

Hemocompatibility limits the intravenous route. Contact of the cell product with blood can trigger IBMIR, affecting safety and the available dose of circulating cells [71]. TF expression on MSCs serves as a trigger for the procoagulant response, with high TF expression being associated with coagulation activation and thromboembolic outcomes [20]. Importantly, however, hemocompatibility is not an intrinsic property of the tissue source but rather a product-specific characteristic determined by multiple variables: TF/CD142 expression levels, cell viability, the presence of aggregates, passage number, culture conditions, washing procedures, formulation, dose, and infusion rate. Therefore, while certain MSC sources may exhibit lower baseline TF expression in comparative studies, routine product-specific hemocompatibility testing is mandatory before intravenous administration to ensure safety and minimize thrombotic risk.

The clinical evidence base for intravenous MSC administration in CNS diseases demonstrates an acceptable safety profile when the product is properly prepared and infusion protocols are followed. In a Phase II study of patients with relapsing-remitting MS, intravenous administration of autologous MSCs was evaluated for safety and was associated with a reduction in the cumulative number of gadolinium-enhancing lesions compared to placebo, which is considered a clinically significant anti-inflammatory effect (NCT01228266) [22].

Other clinical studies have also shown that intravenous MSC administration is feasible and generally well tolerated in MS and amyotrophic lateral sclerosis (ALS), although convincing efficacy for disease activity control has not yet been demonstrated. For MS, this is supported in particular by an open-label Phase IIa study with intravenous infusion of autologous BM-MSCs in secondary-progressive MS (NCT00395200) [72] and a pilot study of intravenous transplantation of autologous MSCs (NCT00813969) [73]. For ALS, direct data on the intravenous route have been obtained from a Phase I study (NCT01759797), published alongside a parallel intrathecal administration group (NCT01771640) [74].

### 4.2. Intrathecal Administration

The intrathecal (IT) route allows delivery of the cell product directly into the cerebrospinal fluid space, bypassing the pulmonary bed and systemic elimination. This increases the likelihood of MSC interaction with cerebrospinal fluid and perivascular structures; however, it is not equivalent to proven integration of cells into the brain parenchyma. The migratory capacity of MSCs is limited—even upon intraventricular administration in animals, cells disperse within a radius of a few millimeters [75]. IT administration is used in diseases with predominantly spinal or cerebellar manifestations, as well as in combination with the intravenous route. In a study on secondary-progressive MS, combined administration was deemed safe and potentially more effective NCT01364246 [76]. From a safety perspective, intrathecal administration carries the risks associated with lumbar puncture, such as post-dural puncture headache, which include headache, back pain, transient fever, and meningeal symptoms [49].

In a Phase II study (NCT03355365) involving intrathecal administration of autologous MSC-derived neural progenitors (MSC-NPs) in progressive MS, tolerability of repeated injections and changes in cerebrospinal fluid (CSF) biomarkers (matrix metalloproteinase (MMP)-9 and chemokine (C-C motif) ligand (CCL)-2) were demonstrated [77]. In ALS, repeated IT administration of BM-MSCs showed a slowing of disease progression with a limited duration of effect, which is consistent with the transient presence of cells in the CSF [78].

### 4.3. Intracerebroventricular Administration

The intracerebroventricular (ICV) route involves administration of the cell product into the ventricular system, providing access to the cerebrospinal fluid compartment and periventricular structures. Ventricular infusions allow for more effective targeting of basal brain regions and brainstem structures compared to lumbar delivery [79].

In a model of severe intraventricular hemorrhage in newborn rats, ICV administration was shown to provide a higher local concentration of MSCs in the brain compared to IV administration. However, functional and morphological neuroprotective outcomes were similar between the groups, suggesting that intravenous administration may be a good alternative for clinically unstable preterm infants for MSC delivery [80]. In a chronic demyelination mouse model, ICV administration of MSCs induced functionally significant remyelination and activation of endogenous processes in the subventricular zone. In a Phase I study in patients with Alzheimer’s disease, three repeated ICV infusions of UCB-MSCs were performed via a reservoir. After each administration, transient fever and elevated CSF white blood cell counts were observed within 24 h; symptoms resolved within 1–2 days, and CSF parameters normalized by week 4 [81,82]. For repeated administrations, a ventricular catheter with a subcutaneous reservoir is used, which adds risks of hemorrhage, infection, malposition, and obstruction [79]. This reaction has been interpreted as transient inflammation associated with administration of the cell product.

Thus, clinical application of the ICV route requires consideration not only of procedural access safety but also of the predictability of the acute CSF immune response to the specific cell product, with the need for protocol-based monitoring. Advantages of this method include controlled delivery to the CSF and the possibility of repeated administrations via an implantable access device. Limitations include the lack of direct parenchymal delivery, risks associated with the catheter–reservoir system, and the potential for transient inflammation in the CSF space.

### 4.4. Intraparenchymal Administration

The intraparenchymal route involves stereotactic implantation of MSCs directly into brain tissue at predetermined coordinates, typically along the periphery of the pathological focus. Cell product exposure is localized within the parenchyma, which fundamentally distinguishes this route from systemic administration in which viable MSCs do not pass beyond the pulmonary capillary bed.

In a clinical study of chronic stroke, implanted cells were shown to persist for approximately 1 month, supporting the interpretation of the effect as being mediated by microenvironmental changes and secreted factors (NCT01287936). In therapy of traumatic brain injury with allogeneic modified BM-MSCs (SB623), the incidence of AEs was high in both groups; however, dose-limiting toxicity was absent, indicating a substantial contribution of the inherent risks of the neurosurgical procedure itself (NCT02416492) [83,84]. In Niemann–Pick disease type C, cerebellar administration of BM-MSCs prevented Purkinje cell loss and reduced sphingoid accumulation [85,86].

From a practical standpoint, the intraparenchymal route is most often chosen in situations where the presumed therapeutic target is localized and stereotactically accessible. Overall, intraparenchymal administration in the context of MSC delivery to the CNS is described as the most direct route. However, the effect should not automatically be attributed to long-term cell presence or integration, and safety represents the combination of risks from the cell product and those of stereotactic neurosurgery [87,88].

A comparative analysis of MSC administration routes shows that delivery to the CNS is always a compromise between access invasiveness, procedural risks, and the controllability of cell product exposure in the target compartment (Table 2). The IV route is characterized by limited biodistribution due to pulmonary entrapment, making direct cell delivery to the brain unlikely and supporting the interpretation of effects as predominantly systemic. Hemocompatibility must be considered, and TF-mediated coagulation risks require control. CSF routes (IT, ICV) increase cell exposure in the CSF compartment, bringing the action closer to the CNS without vascular filtration; however, they do not guarantee parenchymal delivery and carry risks associated with neuraxial access. Intraparenchymal administration is the most direct delivery route in a geometric sense, but it is also the most invasive, and its combination of risks come from the cell product and stereotactic neurosurgery.

## 5. Treatment Regimens: Dosage, Course Frequency, Concomitant Therapy, Premedication, and Monitoring

Even with the same route of administration, the clinical effect and risk profile of MSC therapy are determined by the dose, infusion rate, treatment course frequency, as well as which concomitant medications and safety measures are applied during the course.

### 5.1. Dosing and Administration Frequency

There is no unified therapeutic window for MSCs; dose selection depends on the disease, route of administration, and treatment course frequency [99]. For systemic administration, the dose range varies from 10^5^ to 10^7^ cells/kg, with regimens of 1–10 × 10^6^ cells/kg being most commonly used in clinical protocols [69,99]. For IV administration in neurological indications, dose selection reflects a compromise between achieving a systemic biological effect and ensuring hemocompatibility. For MS therapy (NCT01854957), a dose of 1–2 × 10^6^ MSCs/kg was chosen [100]. In another MS study, a single IV administration of autologous BM-MSCs at a dose of 1–2 × 10^6^ cells/kg was used with no signs of toxicity [73]. For IT administration, doses typically range from 10 to 50 × 10^6^ cells per injection. In a study for ALS therapy, two intrathecal administrations of 1 × 10^8^ cells each were given at a 1-month interval [101]. In another ALS study, two intrathecal injections of autologous BM-MSCs at a dose of 1 × 10^6^ cells/kg were administered 26 days apart [78]. In a randomized controlled trial for cerebral palsy, four IV administrations of 5 × 10^7^ allogeneic UC-MSCs were given at 7-day intervals (ChiCTR1800016554) [102]. For ICV administration, lower doses are used; in a study for Alzheimer’s disease, 1–3 × 10^7^ UCB-MSCs/2 mL were administered three times via an Ommaya reservoir at 4-week intervals (NCT02054208, NCT03172117) [81]. Intraparenchymal implantation is typically performed as a single procedure with a fixed number of cells per lesion. Even when the same route is maintained across studies, not only does the cell number vary but also the volume and concentration of the suspension, infusion rate, and the permissibility of repeated courses—these parameters often determine both safety and reproducibility of biological endpoints [83,87].

Treatment course frequency ranges from a single administration to repeated injections and remains an empirical parameter determined by the expected duration of the biological effect, route invasiveness, and acceptable procedural risks [69]. Most clinical studies lean toward the need for repeated administrations to maintain the therapeutic effect [4]. For example, both single courses and repeated administrations at 3–6 month intervals have been used in MS. Fernández et al. used three intravenous infusions at 3-month intervals in progressive MS with an acceptable safety profile. Some patients received up to eight courses over 5 years [103]. For CSF routes, repeated administrations are used more frequently. In ALS, a cycle of two intrathecal injections at a 1-month interval is used, with the possibility of repetition every 6–12 months (NCT04745299). Another study is testing a regimen of two IT injections (one cycle) followed by booster injections at 4, 7, and 10 months [101].

### 5.2. Concomitant Therapy and Premedication

MSC therapy regimens almost always include pharmacological support, since tolerability of administration and interpretability of endpoints depend on background therapy. In neurological protocols, the study design involves controlling for prior immunotherapy and steroids to minimize confounding effects. Premedication is aimed at reducing the likelihood and severity of acute infusion reactions, which may be caused by either the cell product or formulation components. In an MS study, patients were excluded if they had received immunosuppressive therapy within the preceding 3 months, and the use of IFN-β, glatiramer acetate, or corticosteroids within 30 days prior to randomization was not permitted. For treated patients, premedication consisted of a combination of an antihistamine, an antipyretic, and a glucocorticosteroid (dexchlorpheniramine 2 mg + paracetamol 1 g + methylprednisolone 100 mg) (NCT01228266) [22]. For cryopreserved preparations containing dimethyl sulfoxide (DMSO), antihistamines (chlorphenamine), paracetamol, and hydrocortisone are used, with tolerability depending on the total DMSO dose, infusion rate, and degree of dilution after thawing [104].

For IV administrations, a separate class of measures relates to hemocompatibility and prevention of IBMIR. The procoagulant potential of the product is considered a basis for preclinical hemocompatibility assessment and clinical risk management [53]. In preclinical studies, anticoagulant support with heparin reduced thromboembolic complications and improved cell distribution [105].

Premedication and concomitant therapy are critical determinants of safety and efficacy assessments. Protocols typically restrict immunosuppressants and steroids prior to infusion, whereas antihistamines and antipyretics are administered prophylactically to control infusion-related reactions.

### 5.3. Monitoring and Clinical Regimens in Different Nosologies

Monitoring is aimed toward early detection of infusion reactions, hemodynamic and respiratory disturbances. For example, DMSO can induce histamine release and cause flushing, dyspnea, and abdominal cramps, which justifies observation during infusion and in the early post-infusion period [106]. Protocols include dynamic monitoring of vital parameters before, during, and after administration. In the UCB-MSC infusion protocol, continuous pulse oximetry was applied during infusion and for 1 h after, with vital signs recorded every 15 min during infusion and every 30 min for 1 h after. Protocols specify criteria for immediate discontinuation of infusion if symptoms of an infusion reaction or hypoxemia develop [107]. After MSC administration in MS, patients are monitored for up to 2–6 h [73,100].

The treatment regimen is an independent variable affecting safety and efficacy. In studies of BM-MSCs secreting neurotrophic growth factors (NTF) (NCT01051882) in ALS, single-administration regimens (IT in patients at a more advanced stage) were used, with consideration of progression rates before and after the intervention. The authors reported a slowdown in the rate of decline of the revised ALS functional rating scale (ALSFRS-R) and forced vital capacity (FVC) in some patients compared to the pre-treatment period [108]. In another early Phase 1/2 study involving patients with MS and ALS, a single intrathecal or intravenous administration of autologous MSCs was performed with subsequent long-term follow-up (NCT00781872) [109].

In metachromatic leukodystrophy, a single IV administration of MSCs after bone marrow transplantation demonstrated good tolerability; however, the authors noted the need for multiple infusions to enhance the effect [64]. The cumulative evidence supports multi-stage MSC therapy regimens; for adult patients, 2–3 administrations during the first year followed by effect assessment appears to be optimal.

## 6. Safety Profile: Short-Term and Long-Term Risks

The safety of MSC therapy for CNS diseases hinges on the risks associated with the administration and reactions to the cell product, which may manifest both immediately and in the long term.

### 6.1. Short-Term Risks

Findings from clinical research on MSC therapy in neurology demonstrate the relative safety of the approach; the majority of AEs in the short-term period are mild to moderate in severity and include headache, back pain, and transient fever, with a low incidence of serious complications [93]. Short-term risks vary depending on the route of administration. For the intravenous route, hemocompatibility is of key importance: TF expression on MSCs can trigger procoagulant reactions and IBMIR, necessitating assessment and management of the risk of thrombotic complications [62,71]. Upon IV administration of UC-MSCs to patients with MS, 19 out of 20 participants reported transient fatigue or headache, but no serious adverse events were recorded (NCT02034188) [110]. For the IT route, short-term risks consist of the effects of lumbar puncture and the reaction to administration of the cell product into the CSF. In cerebral palsy, early AEs were predominantly related to post-dural puncture phenomena [111]. In adrenomyeloneuropathy, IT administration of WJ-MSCs was also characterized by acceptable tolerability without severe complications [112].

For intraparenchymal implantation, short-term risks are largely attributable to the neurosurgical procedure itself. In a randomized study (NCT02416492) in chronic post-traumatic deficits, the incidence of adverse events was comparable between groups, with no fatalities reported [88]. Upon ICV administration via an Ommaya reservoir in patients with AD, transient fever was observed after each injection, resolving within 1–2 days, which is interpreted as part of the tolerability of the ICV regimen [81,82]. Infectious risks in the short-term period for CNS routes are primarily associated with invasive access (catheters, reservoirs, repeated punctures) and aseptic technique quality, rather than with the immunosuppressive action of MSCs [109,113].

### 6.2. Long-Term Risks

The long-term safety of MSC therapy is assessed from months to several years and includes tumorigenicity, ectopic differentiation, immunogenicity upon repeated courses, and the risk of adverse effects of trophic factors on disease progression [114,115].

Long-term clinical follow-up generally does not reveal frequent tumor formation directly attributable to MSC administration. For example, in systemic lupus erythematosus, no oncological complications were reported after allogeneic MSC therapy [116]. However, preclinical data indicate a potential capacity of MSCs to promote the growth of existing tumors through microenvironment modulation, which warrants caution in patients with a history of malignancy and strict cell product control in accordance with Good Manufacturing Practice (GMP) standards [117,118]. Factors secreted by MSCs may theoretically potentiate processes that are undesirable in certain nosologies. In oncology, the ability of MSCs to support angiogenesis, immunomodulation, and invasion in tumor models has been discussed [118,119]. In the CNS, similar risks are relevant in the setting of active tumor processes, infection, or uncontrolled inflammation; however, direct clinical evidence of adverse effects of MSC-derived trophic factors for most neurological indications remains insufficient [114,115].

MSCs are capable of participating in ectopic bone formation under certain microenvironmental signals [120]. In the CNS, this risk is considered a low-frequency theoretical concern, requiring long-term neuroimaging surveillance for invasive routes, particularly with intraparenchymal administration [70].

Despite the hypoimmunogenicity of MSCs, cellular and humoral responses to allogeneic cells may develop, including the appearance of anti-donor antibodies, which could potentially reduce the efficacy of repeated administrations. In clinical studies, anti-HLA antibodies have been detected in some patients after allogeneic MSC therapy; however, the clinical significance of this phenomenon requires further evaluation [121]. Upon CNS administration, especially with repeated intrathecal or ICV administrations, immunomonitoring is not always included as a mandatory endpoint [78,122].

### 6.3. Specific Safety Aspects in Lysosomal Storage Disorders

For patients with LSDs, it is critical to distinguish between the safety of administration itself and the risk of misinterpreting the natural course of the disease as therapy-related. In cerebral adrenoleukodystrophy, IT administration of allogeneic MSCs in two patients was feasible and uncomplicated; however, demyelination progression continued [123]. This underscores the need to assess safety and efficacy against the expected natural history of the disease and standards of care.

Within the framework of long-term follow-up of patients with leukoencephalopathy, the key monitored parameters include the dynamics of white matter changes on magnetic resonance imaging (MRI), the appearance or increase of contrast enhancement, newly emerging epileptic seizures, dynamics of CSF parameters upon repeated therapy courses, as well as differential diagnosis between post-injection inflammatory reactions and infectious complications associated with invasive access routes [112].

Beyond safety considerations, the current clinical evidence base suffers from several structural limitations that constrain interpretation of efficacy data [124]. First, most studies are early-phase (Phase I/II) with small sample sizes, lacking statistical power to detect modest but clinically relevant effects. Second, the absence of standardized, harmonized outcome measures across trials severely limits cross-study comparison and meta-analysis. Third, many studies are open-label or single-arm, introducing substantial bias, particularly problematic in diseases with fluctuating symptoms or pronounced placebo responses, such as Parkinson’s disease and multiple sclerosis. Fourth, follow-up durations are generally short, precluding assessment of long-term efficacy and durability of effects. Fifth, patient selection criteria are heterogeneous, with variable disease duration, severity, and prior treatments, which may obscure treatment effects in subgroups who might benefit [103]. Sixth, the lack of validated potency assays that correlate with clinical outcomes means that the administered cell products may vary widely in their functional activity, further diluting treatment effects. Finally, publication bias favoring positive results creates an incomplete and potentially overly optimistic picture of the evidence. These limitations must be addressed in future trial design.

## 7. Evidence Base for MSC Efficacy in Neurodegenerative Diseases

Randomized controlled trials were considered the strongest evidence, whereas Phase I/II, open-label, and single-arm studies were interpreted mainly as evidence of safety and feasibility. Case reports and case series were considered hypothesis-generating, while animal and in vitro studies were regarded as preclinical evidence rather than evidence of clinical efficacy.

Importantly, preliminary signals of clinical improvement should not be interpreted as definitive evidence of efficacy. Early-phase and uncontrolled studies are particularly vulnerable to small sample sizes, a lack of comparator groups, heterogeneous patient populations, and short follow-up, which may limit the reliability and generalizability of observed treatment effects. Therefore, findings from such studies should be considered exploratory until confirmed in adequately powered randomized controlled trials.

### 7.1. Amyotrophic Lateral Sclerosis

The evidence base for MSC-based approaches in ALS has been established primarily around IT administration and repeated courses, aiming to target neuroinflammation and the CNS microenvironment, as direct neuronal replacement is not a realistic goal. This is one of the most extensively studied areas for MSC therapy among neurodegenerative diseases. Several Phase I/II studies have been conducted, demonstrating safety and potential efficacy.

Autologous BM-MSCs demonstrate acceptable tolerability but only modest, time-limited efficacy signals. However, the Phase I study (twice-weekly IT dosing) was designed solely for safety and lacked the power to confirm clinical benefit (NCT01363401) [125]. In a Phase II study with a similar dosing regimen, short-term differences in ALSFRS-R dynamics and concomitant changes in CSF biomarkers were identified; however, sustained confirmation of an impact on long-term outcomes, including survival, was not obtained, indicating a potentially transient nature of the effect, dependent on the administration regimen, treatment frequency, and disease stage [78]. The most rigorous validation of a cell-based platform in ALS was conducted for BM-MSC-NTF in a double-blind, placebo-controlled Phase III study (NCT03280056). The trial confirmed an acceptable safety profile for BM-MSC-NTF administration, with no major safety concerns identified in the treated population. Exploratory analyses revealed biological activity, including changes in CSF biomarkers suggestive of target engagement and modulation of inflammatory pathways; however, despite these biological signals, the primary efficacy endpoint was not met in the overall population, meaning that clinical efficacy according to pre-defined, rigorous criteria in a high-level-of-evidence design was not confirmed at the level of the entire cohort (NCT03280056) [126]. In Phase I/II and IIa studies involving 26 patients, autologous BM-MSC-NTFs were administered via intramuscular and intrathecal routes. In 87% of patients, a slowdown in the decline of motor and respiratory functions was slower than predicted—a finding interpreted as a positive therapeutic response (NCT01051882, NCT01777646) [108]. These results prompted a subsequent study. It found no significant slowing of progression in the overall group; however, post hoc analysis showed improvement in rapid progressors (NCT04745299). On this basis, in South Korea, the product Neuronata-R (lenzumestrocel) received conditional approval as an orphan drug for IT administration in ALS [101]. These findings illustrate the importance of distinguishing exploratory subgroup signals from prospectively defined primary efficacy outcomes, particularly when interpreting early-phase or negative randomized trials.

### 7.2. Alzheimer’s Disease

In preclinical models of AD, MSC therapy has been studied in both acutely induced and transgenic mouse models. ICV administration of BM-MSCs into the hippocampus in an acute β-amyloid (Aβ)-induced model in C57BL/6 mice reduced Aβ levels and was accompanied by microglial activation near amyloid deposits, consistent with enhanced microglial clearance of Aβ [127]. In another study, IV administration of MSCs derived from the amniotic membrane of the placenta to transgenic C57BL/6J-APP mice improved learning and memory, as well as reduced amyloid burden in the cortex and hippocampus [128].

Clinical data on the use of MSCs in AD are at an accumulation stage and are represented predominantly by early-phase studies and small randomized trials with a focus on safety and biomarkers; convincing clinical efficacy with respect to cognitive outcomes has not been demonstrated overall. Accordingly, changes in biomarkers or exploratory clinical measures should be regarded as preliminary signals rather than evidence of established therapeutic efficacy.

In a Phase I study, patients with AD received ICV administration of UCB-MSCs at 4-week intervals via an Ommaya reservoir. The intervention proved feasible and tolerable, though each injection was followed by a transient fever that resolved within 1–2 days (NCT02054208) [81]. In a subsequent analysis, which included clinical data and a transgenic model, ICV administration of UCB-MSCs was associated with a transient inflammatory response in the CSF and alterations in the cytokine profile, which is significant for interpreting safety and biomarker endpoints upon repeated administrations [82]. In a Phase IIa study, ICV administrations at 4-week intervals did not reveal significant clinical benefits; however, changes in CSF biomarkers of AD were recorded compared to placebo. A higher number of adverse events, such as fever, headache, nausea, and vomiting, were noted in the MSC group; in the open-label phase, the addition of dexamethasone was considered as an approach to reducing immune-mediated reactions and CSF leukocyte/IL-6 levels (NCT03172117, NCT04954534) [129].

With IT administration of autologous BM-MSCs every 3 months, the authors reported stabilization of condition, symptom improvement in some AD patients, and increased cerebral metabolism. In the absence of a control group and the inability to separate the effect of the intervention from natural variability, these data represent an observational signal [130]. Another study assessed the safety of intravenous administration of BM-MSCs and its effect on disease progression and brain atrophy. The results demonstrated a slowing of brain volume decline and a trend toward stabilization of cognitive function (NCT05233774) [131].

### 7.3. Parkinson’s Disease

In preclinical models, intrastriatal (intraparenchymal) administration of MSCs promoted restoration of dopamine levels, improvement of motor functions, and an increase in the number of tyrosine hydroxylase (TH)-positive neurons, which have been attributed to the survival of endogenous neurons under the influence of paracrine factors, as well as to the activation of migration of endogenous neural progenitors to the site of injury [132].

Clinical studies of MSCs in PD have long been represented predominantly by small uncontrolled studies with intracerebral (intraparenchymal) delivery, demonstrating feasibility and acceptable safety with limited evidence of efficacy. With a single administration of autologous BM-MSCs into the sublateral ventricular zone, improvement in unified PD rating scale (UPDRS) scores was described in some patients, with no serious AEs [97]. In another pilot study, bilateral transplantation of allogeneic BM-MSCs into the subventricular zone was associated with UPDRS improvement only in the early-stage PD group [133]. Upon IV infusions of allogeneic BM-MSCs, in the group receiving three administrations, the proportion of patients achieving clinically significant UPDRS improvement was higher than in the placebo group at a specified time point. However, the mean changes in motor scales were characterized by a pronounced placebo effect, and the two-infusion regimen was inferior to placebo in several comparisons, underscoring the sensitivity of results to dosing regimen and the complexity of interpreting clinical scales in PD. With respect to safety, mild transient events predominated; panel-reactive antibodies, interpreted as donor-specific, were detected in some patients, which is significant for repeated courses of allogeneic therapy [134].

In recent years, there has been a shift toward the use of MSC-derived exosomes. In preclinical models, UC-MSC-derived exosomes administered to 6-hydroxydopamine-stimulated SH-SY5Y cells reduced motor deficit severity and dopaminergic neuronal death, presumably through induction of autophagy and the ability to cross the BBB [135]. AD-MSC-derived exosomes enriched with miR-188-3p in 1-methyl-4-phenyl-1,2,4,5-tetrahydropyridine (MPTP)-induced mouse models of PD modulated inflammatory and oxidative pathways, improving behavioral and histological outcomes [136]. It is important to distinguish between these complementary but methodologically distinct lines of evidence: the in vitro SH-SY5Y experiments demonstrate direct cytoprotective effects on dopaminergic-like cells under oxidative stress, whereas the in vivo rodent studies provide evidence of functional motor recovery and histological preservation in a whole-organism context. Clinical studies of MSC-derived EVs in PD are in early-stage trials (NCT06607900, NCT05152394) [137].

### 7.4. Multiple Sclerosis

In MS, an autoimmune demyelinating disease, both immunomodulation and neuroregeneration are of key importance. The cumulative results of clinical studies indicate possible anti-inflammatory and neuroprotective activity of MSCs; however, the magnitude and reproducibility of the clinical effects remain limited, and interpretation depends significantly on the disease phenotype, selected endpoints, regimen, and route of administration.

In a study with a single intravenous administration of autologous BM-MSCs (1 × 10^6^/kg), at six months, some patients showed improvement in vision, while others demonstrated stabilization of Expanded Disability Status Scale (EDSS) scores [73]. In another study, seven IV administrations of allogeneic UC-MSCs over one year showed improvements in pelvic organ function, walking, and quality of life (NCT02034188) [110]. In another study, combined IT and IV MSC administration over a 10-year follow-up period was not associated with serious AEs [76]. While these observations suggest potential biological activity, they derive from small, uncontrolled studies and should be interpreted as hypothesis-generating rather than confirmatory evidence of efficacy.

In relapsing-remitting MS, a Phase II study evaluated intravenous infusion of autologous BM-MSCs (NCT01854957). The results did not meet the primary efficacy endpoint but demonstrated a favorable safety profile with no infusion-related serious AEs and no fatalities [138]. Another study assessed the cumulative number of gadolinium-enhancing lesions at six months following intravenous administration of BM-MSCs; no serious AEs were observed, though the efficacy signals were modest (NCT01228266) [22]. Thus, in active MS, intravenous MSC administration has demonstrated an acceptable safety profile, but convincing efficacy in reducing disease activity has not been established in rigorously controlled settings.

For secondary-progressive MS, intravenous administration of autologous BM-MSCs was evaluated using structural and functional measures of the visual pathway. Observed improvements in several visual parameters are indicative of possible neuroprotection, though the clinical relevance and durability of these effects remain uncertain (NCT00395200) [72]. IT administrations of MSC-NPs showed improvement in median EDSS scores, with observed improvement in muscle strength and bladder function; however, these findings are derived from an open-label study without a placebo control, limiting the strength of the conclusions (NCT01933802) [122].

### 7.5. Other Diseases

Clinical data on MSCs in other neurodegenerative diseases are largely limited to early phases, small sample sizes, and a focus on safety and biomarkers.

For multiple system atrophy (MSA), the safety and tolerability of autologous AD-MSC administration have been confirmed, with a painful reaction noted at higher doses and dose-dependent signals on clinical scales discussed as a rationale for further investigation [92].

For progressive supranuclear palsy, intra-arterial administration of autologous BM-MSCs improved dopaminergic function in preclinical and clinical models (NCT01824121) [139]. In a pilot study, intra-arterial MSC administration led to stabilization of degeneration for at least 6 months (NCT01824121) [140].

In spinocerebellar ataxias (SCA), the use of intrathecal UC-MSCs has been described, with improvements or slowing of progression on clinical scales (NCT03378414). Patients with SCA and the cerebellar variant of multiple system atrophy (MSA-C) received IT UC-MSCs at a dose of 1 × 10^6^/kg, for a total of four administrations per course. At 1 month after treatment, scores on the International Cooperative Ataxia Rating Scale (ICARS) and the Activities of Daily Living (ADL) scale improved significantly, with reductions in gait and standing instability, slowness of movement, fine motor impairment of the upper limbs, writing difficulties, and dysarthria [141].

To facilitate comparison of the available evidence, clinical and translational studies are summarized in Table 3 according to study design, MSC source, route of administration, main outcomes, and key limitations.

## 8. Evidence for MSC Efficacy in LSD

MSCs are being investigated as an adjunctive therapy for LSDs, with evidence largely restricted to preclinical data and limited clinical observations. The available findings primarily derive from animal models and isolated case reports, in which MSCs provide enzyme cross-correction, immunomodulation, and reduction of substrate accumulation.

### 8.1. Krabbe Disease

Krabbe disease is an autosomal recessive neurodegenerative disorder caused by deficiency of the lysosomal enzyme galactocerebrosidase, which is responsible for degradation of galactocerebrosides and sphingolipids that are abundant in myelin membranes [142]. As a result of the disease, incompletely metabolized galactocerebroside accumulates, leading to progressive and severe demyelination. Transplantation of BM-MSCs and AD-MSCs into the brains of mice modeling Krabbe disease, which recapitulate the full spectrum of the disorder’s key pathological and clinical features, resulted in detectable cell persistence and expression of markers associated with neural cell types (including astrocytes, neurons, and oligodendrocytes) without tumor formation. This demonstrated the capacity of MSCs to integrate into the affected brain [39,143]. In a study by Ripoll et al., the effects of ICV administration of BM-MSCs and AD-MSCs were evaluated in a mouse model; both MSC types led to a marked reduction in the CNS inflammatory response and slowed myelin loss [143]. These findings indicate that MSCs can act as anti-inflammatory agents by reducing microglial activation during disease progression.

### 8.2. Mucopolysaccharidoses

Mucopolysaccharidoses (MPSs) are a group of congenital metabolic disorders caused by deficiencies in the lysosomal enzymes required for the breakdown of glycosaminoglycans (GAGs). GAGs progressively accumulate in multiple tissues, leading to multisystem damage, including irreversible changes in the central nervous system and the musculoskeletal system [144]. Most types of MPS, with the exception of MPS IV and VI, primarily involve the central nervous system. Among the various MPS types, the pattern of CNS involvement is highly variable: MPS I (particularly the severe Hurler phenotype), MPS II (Hunter syndrome), MPS III (Sanfilippo syndrome), and MPS VII (Sly syndrome) are characterized by significant neurocognitive impairment and progressive neurological deterioration, whereas MPS IV (Morquio syndrome) and MPS VI (Maroteaux-Lamy syndrome) typically lack primary neurocognitive involvement, with disease manifestations predominating in the skeletal and connective tissues [145]. For certain forms of MPS, such as MPS I and MPS VI, hematopoietic stem cell transplantation (HSCT) is established mainly for severe MPS I, while for MPS VI, ERT with galsulfase is the standard disease-specific therapy, and HSCT has been explored or used in selected settings [146,147]. However, HSCT does not always fully correct the clinical features of the disease, and its benefits are most pronounced when initiated before the onset of significant neurological damage [148,149]. Against this background, MSCs, with their potential to enhance the effects of HSCT, are being considered as a promising adjuvant or alternative approach.

MPS VII, caused by deficiency of b-glucuronidase, is a rare form of the disorder. Among its characteristic and difficult-to-treat manifestations are corneal clouding, developmental delay, and hepatosplenomegaly, which are not corrected by HSCT. Coulson-Thomas et al. demonstrated an alternative local cell therapy strategy. The investigators injected UCB-MSC directly into the corneal stroma of mice with MPS VII. The results showed that the transplanted MSCs not only engrafted in the tissue but also actively participated in the catabolism of accumulated GAGs, suggesting that a similar approach could be applied to treat corneal disease in humans [150].

MPS II and MPS III represent the greatest therapeutic challenge due to early and severe CNS involvement. The efficacy of HSCT in MPS II remains controversial [151]. To date, no MSC-based therapies exist for MPS II or MPS III, but preclinical work is actively progressing.

### 8.3. Niemann–Pick Disease

Niemann–Pick disease (NPD) is a group of inherited autosomal recessive disorders classified as lysosomal storage diseases. Traditionally, NPD is divided into types A and B, caused by acid sphingomyelinase deficiency, and types C and D, which result from mutations in either the *NPC*1 or *NPC*2 gene, leading to disrupted intracellular trafficking of cholesterol and sphingolipids [152]. Cellular therapy, particularly the use of MSCs, is considered a promising strategy that targets the pathogenic mechanisms of the disease. BM-MSCs have demonstrated the ability to fuse with Purkinje cells following intracerebellar administration. Such cell fusion resulted in viable binucleated cells that exhibited increased survival compared with affected neurons [85]. This process is hypothesized to allow donor MSCs to directly deliver functional components into neurons, including potentially the wild-type protein encoded by *NPC*1. This has led to the proposal of a combined strategy in which MSCs genetically modified to express *NPC*1 could selectively deliver the gene to the most vulnerable neuronal populations. It has also been shown that transplantation of BM-MSCs into the cerebellum of mice with NPD reduces neuronal accumulation of sphingolipids, increases neuronal survival, and reduces apoptosis [86]. These results demonstrate that the therapeutic action of MSCs is not limited to simple fusion but also encompasses active modulation of intracellular metabolism [153].

MSCs can modulate the brain microenvironment by reducing neuroinflammation. In NPD, activated microglia and astrocytes release pro-inflammatory cytokines, which significantly exacerbate neurodegeneration [154]. In mouse models of NPD, transplantation of BM-MSCs has been shown to prevent Purkinje cell death in the cerebellum and reduce levels of the toxic sphingosine. These findings indicate that MSCs can directly modulate metabolic pathways disrupted in LSDs [86]. Transplanted MSCs have demonstrated the ability to restrain glial hyperactivation, reducing overall neuroinflammation and creating a more favorable environment for neuronal survival, which supports their consideration as a therapeutic option for treating neurodegenerative diseases.

### 8.4. GM1 and GM2 Gangliosidoses

Gangliosidoses are a group of LSDs characterized by excessive accumulation of the gangliosides GM1 and GM2. GM1 gangliosidosis presents with both central nervous system and systemic manifestations, whereas GM2 gangliosidosis primarily affects the CNS. Despite differences in genetic etiology and biochemical defect, both disorders are inherited in an autosomal recessive manner and share a spectrum of clinical presentations ranging from a severe infantile form to milder, chronic adult-onset variants [155].

Knockout mice deficient in β-galactosidase, representing an authentic disease model, were used in the study. The investigators transplanted BM-MSCs expressing human β-galactosidase into the brain ventricles of neonatal recipient mice. The transplanted cells effectively migrated into the brain parenchyma and persisted for at least 8 weeks, accompanied by a reduction in GM1 ganglioside levels in brain tissue. However, by 6 months after transplantation, the MSCs were no longer detectable in the recipients’ brains, indicating limited long-term cell survival. Achieving a durable therapeutic effect in the CNS will require the development of methods to avoid immune rejection of the transplanted cells or to induce tolerance to the therapeutic gene product [156].

For Tay–Sachs and Sandhoff diseases (GM2-gangliosidoses), no direct clinical applications of MSCs have been described. Nevertheless, a study in a mouse model of Sandhoff disease demonstrated co-transplantation of neural progenitor cells and MSCs. This combination resulted in increased animal survival and partial metabolic cross-correction. The HexB enzyme produced by the transplanted cells was actively taken up by brain microglia, leading to a statistically significant reduction in accumulated GM2-ganglioside levels [157]. This preclinical study is directly relevant to the therapeutic potential of MSCs.

The principle of enzyme delivery via derivatives of transplanted cells is fully applicable to MSCs as well. It has been shown that genetically modified MSCs overexpressing the deficient enzyme exhibit high cross-correction efficiency in vitro and are considered a promising option for combination therapy of LSDs. Furthermore, upon IV administration of genetically modified MSCs to rats, no immune response against the transplanted cells was observed, while HexA activity was detectable in the animals’ tissues [5]. Collectively, these findings provide a preclinical rationale for the further development of cell-mediated gene therapy for GM2-gangliosidoses, combining the immunomodulatory properties of MSCs with the efficacy of genetic correction of enzyme deficiency.

### 8.5. Metachromatic Leukodystrophy

Metachromatic leukodystrophy (MLD) is caused by a defect in arylsulfatase A (ARSA), a key enzyme in the catabolism of myelin-enriched sphingolipids. Progressive accumulation of sulfatides leads to severe demyelination and neurodegeneration of the peripheral nervous system and CNS [158]. The disease primarily affects the myelin sheath of nerve fibers, resulting in progressive motor and cognitive decline. Patients exhibit gait disturbances or delayed acquisition of early motor milestones, followed by speech regression, peripheral neuropathy, ataxia, and visual and auditory impairments [159].

In a subsequent expanded study, the authors enrolled six patients with MLD and administered expanded MSCs intravenously without prior ablation. The procedure demonstrated a favorable safety profile and no significant immunoreactivity, despite the use of calf serum during culturing. Importantly, four of the six patients showed improved nerve conduction velocity, which was regarded as a possible sign of remyelination. Based on these data, the authors proposed intrathecal administration for targeted delivery to the CNS, as well as combining MSCs with HSCT [64].

The dual-action concept was confirmed in a clinical case. In an adult patient with a slowly progressive form of MLD, the combination of HSCT and two sequential MSC administrations led to stabilization of neurological status over a 40-month follow-up period. Notably, ARSA activity normalized by the 16th month after therapy [42]. This case highlights the key advantage of the combined approach: HSCT ensures sustained enzymatic recovery, whereas MSCs exert neurotrophic support and immunomodulation, thus establishing conditions that restrain disease progression.

A study was conducted summarizing the experience of combined therapy in 10 children with MLD. According to the protocol, patients underwent allogeneic HSCT followed by two administrations of MSCs at a dose of 2 × 10^6^/kg body weight on days 30 and 60. The results confirmed the long-term safety of the approach: adverse events potentially related to MSCs were not observed over up to 13.5 years of follow-up. In the combined therapy group, a statistically significant increase in ARSA activity was observed, along with higher white blood cell and platelet counts during the first year post-transplantation, indicating improved engraftment and hematopoiesis [160].

The gap between preclinical promise and clinical translation is particularly pronounced in LSDs, where the majority of evidence comes from murine models that do not fully recapitulate the human disease. While knockout mouse models of LSDs have been invaluable for understanding disease mechanisms, they often fail to reproduce the full spectrum of human pathology, including the characteristic neurodevelopmental and neurodegenerative features. For example, in many LSD mouse models, the CNS phenotype is milder or develops more slowly than in humans, limiting the ability to assess therapeutic efficacy on clinically relevant outcomes [161]. Additionally, the timing of intervention in preclinical studies frequently at birth or in early postnatal life is rarely achievable in human patients, who are typically diagnosed after significant neurological damage has occurred. This discrepancy in timing likely contributes to the limited efficacy of MSCs observed in clinical settings, where the therapeutic window may have already closed. Furthermore, the physical size of the rodent brain allows transplanted MSCs to achieve relatively widespread distribution, whereas the much larger human brain presents an insurmountable barrier to cell dispersion from focal injection sites. These considerations suggest that, for LSDs, the primary therapeutic contribution of MSCs may lie in their immunomodulatory and neurotrophic support functions, rather than in direct enzyme delivery or cross-correction, and that combination approaches with gene or enzyme replacement therapy may be necessary to achieve clinically meaningful benefit [4].

## 9. MSC-EVs: An Alternative to Cell Therapy

The therapeutic potential of MSCs in LSDs is largely mediated by their secretion of biologically active molecules rather than by direct replacement of defective cells. In this context, interest in recent years has shifted toward using not MSCs themselves but their exosomes—nanoscale extracellular vesicles. By recapitulating the key therapeutic effects of the parent cells, EVs offer several advantages in terms of safety, controllability, and logistics [162].

### 9.1. Characterization of MSC-EVs and Mechanisms of Action

MSC-EVs are membrane vesicles 30–150 nm in size, which carry proteins, lipids, nucleic acids, microRNAs, and other regulatory molecules [6]. Due to their small size, MSC-EVs may cross or interact with the BBB under certain conditions, which could represent an advantage for the treatment of neurodegenerative diseases—provided that biodistribution is favorable depending on route, source, engineering, disease state, and dose. Once in the brain parenchyma, they deliver their contents to target cells, modulating their metabolism, inflammatory response, and survival [6].

The therapeutic potential of MSC-EVs in LSDs is realized through several mechanisms. Firstly, they carry a wide range of neurotrophic factors and anti-inflammatory cytokines that promote neuronal survival and reduce the activity of pro-inflammatory microglia. Secondly, they may contain microRNAs capable of regulating neurogenesis, synaptic plasticity, and apoptosis. In the treatment of neurodegenerative diseases, overexpression of miR-188-3p in exosomes derived from AD-MSC had a neuroprotective effect in a PD model [136]. In an AD study, IV administration of MSC-EVs led to a decrease in the level of Aβ plaques in the brain and improved cognitive functions in mice, which is associated with the delivery of Aβ-cleaving enzymes and anti-inflammatory microRNAs [163]. Thirdly, MSC-EVs can modulate microglial activity, shifting their phenotype from pro-inflammatory to anti-inflammatory, which is important for alleviating neuroinflammation common to many LSDs. This effect is relevant to a wide range of conditions. For example, in experimental spinal cord injury, the introduction of MSC exosomes significantly improved motor functions and stimulated axon regeneration [164]. In another study on a model of traumatic brain injury in rats using AD-MSC-EVs, it was shown that small EVs (sEVs) injected into the brain ventricles selectively accumulate in the injury zone and exhibit pronounced colocalization with microglia [165].

The most promising approach for the treatment of LSDs is the use of EVs as a platform for targeted delivery of therapeutic agents. Current technologies allow EVs to be loaded with specific cargo ex vivo. It has been demonstrated that EVs loaded with α-galactosidase effectively deliver the enzyme to target cells in Fabry disease, resulting in reduced accumulation of the pathological substrate [166]. More broadly, strategies for enhancing the therapeutic potential of EVs can be divided into two main categories: endogenous and exogenous cargo loading. The endogenous approach involves genetic modification of producer cells to overexpress target therapeutic molecules, which are then naturally packaged into secreted EVs. This method is more preferable in terms of reproducibility. The alternative, the exogenous approach, involves directly introducing drugs into EVs. Common methods include electroporation, which creates temporary pores in the EV membrane using high-voltage pulses; incubation, where the drug enters the EVs under a concentration gradient; sonication; cyclic freeze–thawing, which disrupts membranes; extrusion through narrow channels; and chemical transfection. The choice of the best loading method depends on the EV source, cargo, and intended application. However, active loading methods can cause aggregation of small RNAs, disruption of membrane structures, loss of EVs, and reduced product purity [167]. Despite the effectiveness of this approach, its development can be complemented by the use of MSC-derived EVs, which possess immunomodulatory properties that may potentially enhance the therapeutic effect. MSC-EV-based products are closest to clinical implementation and industrial production, compared to EVs derived from other cells.

### 9.2. Advantages of MSC-EVs

The transition from cell therapy to EV therapy is driven by several advantages that address key challenges in the clinical application of MSCs.

EVs, being anucleate structures, lack the ability for uncontrolled proliferation, spontaneous differentiation, or malignant transformation, thereby eliminating the risk of tumor formation. Major histocompatibility complex (MHC) molecules are virtually absent on their membrane, making MSC-EVs minimally immunogenic and allowing the creation of allogeneic products without the need for donor matching or immunosuppression [168].

**Figure 3:**
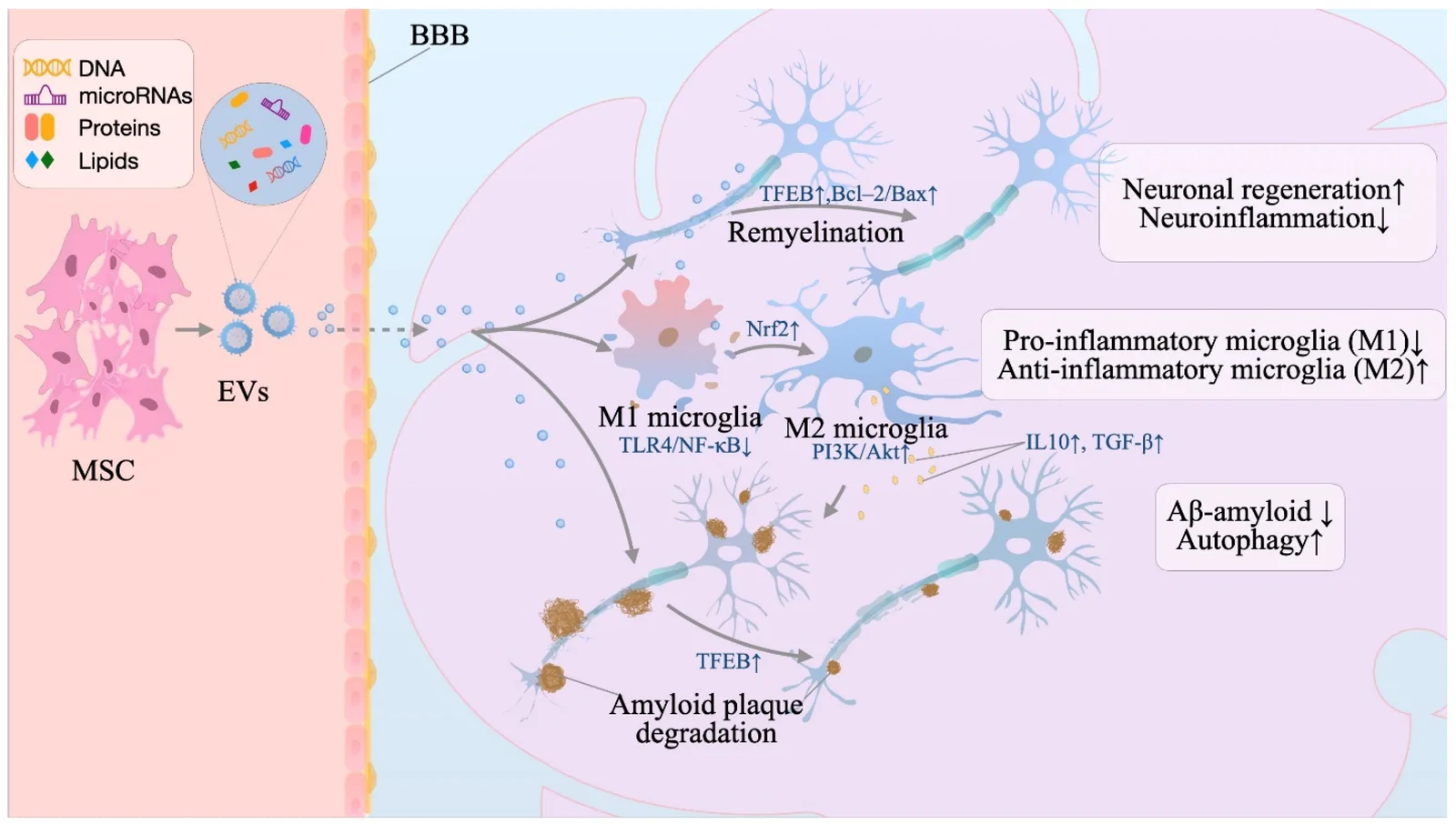
Neuroprotective mechanisms of mesenchymal stromal cell extracellular vesicles in neurodegenerative diseases.

The main advantage of EVs in the therapy of neurodegenerative diseases is that upon peripheral administration (including intravenous, intrathecal, and intraperitoneal routes), EVs can deliver therapeutic agents by crossing the BBB and reaching the brain parenchyma, whereas whole MSCs are largely retained in the microvasculature of the lungs and liver (Figure 3) [169]. However, the biodistribution pattern of MSC-EVs depends significantly on the route of administration. Intravenous administration results in predominant accumulation in the abdominal cavity, whereas intranasal administration provides localization directly in brain tissues, and intratracheal administration leads to lung accumulation [170]. Local routes of administration can significantly increase the concentration of MSC-EVs in target organs while limiting their systemic distribution, which may be particularly advantageous for the treatment of neurodegenerative diseases.

To improve their delivery efficiency, surface modification strategies for EVs are being developed to enable active targeting. For example, EVs derived from MSCs pretreated with iron oxide nanoparticles form magnetic nanovesicles. These can be magnetically guided to ischemic brain regions under an external magnetic field and contain large amounts of therapeutic growth factors [171]. Conjugation of the cyclic peptide (Arg-Gly-Asp-D-Tyr-Lys) [c(RGDyK)] to the EV surface provides high-affinity binding to reactive brain vascular endothelial cells containing c(RGDyK) and the αvβ3 protein, thereby targeting migration to the ischemic brain region [172]. It is important to emphasize that excessive surface modification can lead to risks such as increased immunogenicity or unintended changes in biodistribution [167].

MSC-EVs are smaller and less complex than living cells, which simplifies their production, standardization, cryopreservation, and transport. They have lower requirements for storage conditions, making them more practical for widespread use [173].

Both MSCs and their EVs express TF and demonstrate measurable procoagulant activity in vitro [20]. However, the risk of thrombogenic events with MSC-EVs is considered significantly lower compared to whole MSCs, due to their smaller size, absence of a cellular surface, and different pattern of interaction with blood cells and endothelium. Nevertheless, this aspect requires mandatory monitoring in clinical applications.

### 9.3. Challenges in the Production of MSC-EVs

Despite their significant potential, the transition to widespread clinical application of MSC-EVs requires solving several technological challenges arising from the product’s characteristics. First, the productivity of MSCs in culture is limited; it is advisable to use early-passage cells for exosome production, which is associated with the preservation of their functional properties [174]. Low exosome yield per cell and the lack of standardized industrial platforms for their production remain major barriers to clinical scaling [175]. Currently, various culture platforms are used for the production of MSC-EVs. Conventional two-dimensional culture in tissue-culture flasks remains the most widely used approach, particularly at the laboratory scale. However, it is labor-intensive, requires a large surface area, and has limited scalability. Three-dimensional culture systems, including hollow-fiber bioreactors, provide a higher cell density and a more controlled culture environment. These systems may increase EV productivity by promoting physiologically relevant cell–cell interactions and improving nutrient and gas exchange [175].

A critical step in the production of therapeutic EVs is the removal of contaminating components, including cellular debris, apoptotic bodies, and protein aggregates [176,177]. Differential ultracentrifugation remains one of the most widely used methods for EV isolation. However, this method is labor-intensive and may result in the co-isolation of soluble proteins and other contaminants. Several alternative approaches are also used, including ultrafiltration, size-exclusion chromatography, immunoaffinity chromatography, and polymer precipitation. Each method has specific advantages and limitations with respect to EV recovery, purity, processing time, scalability, and reproducibility. The most effective approach is recognized to be the combination of several methods to achieve an optimal balance of purity, yield, and reproducibility [176,177]. Second, the composition and functional properties of EVs vary depending on the MSC source, culture conditions, passage number, and isolation method. To obtain a reproducible therapeutic product, the implementation of strict characterization protocols is required, including analysis of particle size and concentration, surface markers, as well as proteomic and microRNA profiling to assess product purity [20,178]. The most common markers are CD9, CD63, CD81, and CD82, MHC molecules, heat shock proteins (HSPs), and tumor susceptibility gene 101 (Tsg101) [179]. For clinical application, demonstrating product stability and reproducibility of functional properties is critical, which necessitates validation of isolation and storage methods, as well as strict control of culture conditions for producer MSCs [178].

### 9.4. Current Status of MSC-EVs

To date, the main research on MSC-EVs has been focused on oncology (as drug delivery systems) and regenerative medicine [168]. Early clinical studies on MSCs and their EVs in severe COVID-19 suggest feasibility, safety, and possible immunomodulatory effects, though the evidence is heterogeneous and not directly transferable to CNS or LSD settings. The results of numerous clinical trials have shown that MSC-EVs therapy leads to a significant reduction in pro-inflammatory cytokine levels, improved oxygenation, and shortened recovery time in patients with acute respiratory distress syndrome [180]. These data confirm the immunomodulatory and regenerative potential of MSC-EVs in clinical settings.

In neurology, MSC-EV-based therapies remain at an early stage, while their application to LSDs is even less advanced and is currently supported mainly by preclinical evidence. In 2024, one of the world’s first Phase I clinical trials of MSC exosomes for PD was initiated (NCT05152394). In addition, clinical trial registries include studies of MSC-EVs in AD (NCT04388982), multiple sclerosis (NCT07146087), and ischemic stroke (NCT06995625), though most are in Phase I–II with small cohorts, where the primary objective is safety assessment rather than efficacy. The results of these studies may serve as a starting point for neurology and for extending this technology to other neurodegenerative diseases.

Thus, MSC-EVs represent a promising alternative to MSCs. They retain the key therapeutic mechanisms of action of MSCs (immunomodulation, neuroprotection, and potential for metabolic correction) while offering a substantially improved safety and manageability profile.

## 10. Proposed Individualized Monitoring Framework: Scales, Biomarkers, and Neuroimaging

Monitoring patients with neurodegenerative diseases requires a comprehensive approach combining clinical assessment, laboratory biomarkers, and instrumental methods. The optimal monitoring strategy is built around key time points: baseline assessment before therapy initiation, intermediate monitoring every 3 months, and evaluation at 6–12 months, based on which a decision is made regarding repeated treatment courses [92,181].

First and foremost, a general assessment of the patient’s condition must be performed before treatment initiation. The baseline assessment serves as the foundation for all subsequent comparisons. A neurological examination is performed with detailed documentation of all symptoms and their severity. Clinical assessment is based on the Scale for the Assessment and Rating of Ataxia (SARA) as a core scale for quantitative assessment of cerebellar ataxia; the International Cooperative Ataxia Rating Scale (ICARS) may be used as a more detailed alternative to SARA (NCT00202397) [182]. For global assessment of disability and functional independence, the modified Rankin Scale (mRS) and the Barthel Index are used, while the Gross Motor Function Classification for MLD (GMFC-MLD) may be applied in cases of MLD [183]. If cognitive complaints are present, screening scales are included in the baseline assessment: the Montreal Cognitive Assessment (MoCA) or the Mini-Mental State Examination (MMSE) [184]. In a clinical study on AD, MoCA was used for screening and for assessing cognitive dynamics during the intervention, helping to identify a statistically significant improvement optionally, the Trail Making Test (TMT) may be used to assess information processing speed, visuomotor skills, sequencing, and cognitive flexibility [185].

### 10.1. Laboratory Biomarkers

Laboratory examination before therapy initiation aims to establish baseline levels of key biomarkers. Since MSC therapy is aimed at modulating the microenvironment, priority is given to biomarkers of inflammation and secondary changes. The level of neurofilament light chain (NfL) in blood plasma is determined as a universal marker of neurodegeneration; stabilization of its level or slowing of its increase would be a positive signal for the patient [186,187]. To assess the baseline inflammatory status, pro-inflammatory cytokines (IL-6, TNF, IL-10, etc.) are measured, which is important for subsequent confirmation of the immunomodulatory effect of MSCs [78]. Concurrently, disease-specific biomarkers are assessed: in MPS I, GAG levels in urine and CSF are determined [188], in Tay-Sachs and Sandhoff diseases, HexA activity and GM2 accumulation are measured [189], in MLD, sulfatide isoforms are assessed [158].

The standard safety panel includes a complete blood count (CBC) and biochemical profile, which are necessary to assess the patient’s general condition and to document the hematological phenotypes characteristic of LSDs that may be present at baseline [190,191]. For instance, at the time of diagnosis, abnormalities in red blood cells, leukocytes, and lymphocytes are often noted. For example, vacuolated lymphocytes are observed in GM1 gangliosidosis and MPS III, while foamy macrophages are found in NPD [192].

Since experimental studies have shown that MSCs may promote tumor neovascularization through the secretion of VEGF and other cytokines, exploratory assessment of plasma VEGF levels may be considered as an optional safety marker in the laboratory monitoring plan. Baseline measurement and monitoring at 3, 6, and 12 months could provide ancillary information regarding the absence of pathological angiogenesis activation as a potential mechanism of adverse events.

### 10.2. Neuroimaging

Instrumental examination begins with MRI of the brain and, when clinically indicated, the spinal cord. MRI is an essential tool for detecting profound white matter demyelination characteristic of many LSDs [193]. In later stages, MRI shows progressive atrophy of various brain regions; for example, cerebellar and basal ganglia atrophy is typical in gangliosidoses, while in NPD, atrophy is more pronounced in the frontal lobes [193,194]. The rate of atrophy serves as a marker of disease progression [195].

Associated changes in brain metabolism can be quantitatively assessed using magnetic resonance spectroscopy (MRS) and may provide additional information as biomarkers for disease characterization, progression, and regression. The most clinically significant biomarker of axonal integrity is the N-acetylaspartate to creatine ratio (NAA/Cr). A study involving patients with MLD demonstrated a strong correlation between NAA levels and clinical parameters. These data confirm that NAA is the most clinically significant biomarker of axonal integrity in neurodegenerative diseases with demyelination [196]. In Tay-Sachs disease research, a decrease in the NAA/Cr ratio was documented, reflecting neuronal loss; additionally, an increase in the choline to creatine ratio (Cho/Cr) and elevated myoinositol levels were recorded, indicating gliosis and neuroinflammation [197]. Stabilization of the NAA/Cr ratio at 6–12 months after therapy initiation may indicate a neuroprotective effect of MSCs. Including MRI with MRS in the monitoring plan allows for objective assessment of therapeutic effects.

Intermediate monitoring at 1–3 months is necessary to assess AE and early signs of efficacy, as well as to differentiate a transient post-infusion response from a true therapeutic effect. One month after administration, a complete blood count and biochemical profile are performed to rule out hematological abnormalities and assess VEGF dynamics. In one study, intracerebroventricular infusions of UCB-MSCs for AD involved three administrations at 4-week intervals, accompanied by CSF biomarker monitoring on day 1 after each infusion and at 4 weeks, as well as clinical assessment at 12 weeks after the first administration [81]. In a study on NPD, a 12-week interval demonstrated sufficient sensitivity of the SARA scale to detect significant changes in clinical status: the mean score change was −1.97 ± 2.43 in the treatment group versus −0.60 ± 2.39 in the placebo group [198]. This justifies the choice of a three-month interval for clinical assessment after initiation of MSC therapy.

The inflammatory microenvironment can lead to increased expression of MHC class II on MSCs and enhance their immunogenicity. Furthermore, the presence of induced alloantibodies against a previous infusion is associated with reduced survival of repeatedly administered cells [199]. Thus, it is advisable to assess pro-inflammatory cytokine levels before the second course [78].

For instrumental assessment, follow-up brain MRI is performed (at 6 and 12 months) [112]. Quantitative comparison of cerebellar volume, corpus callosum thickness, and ventricular width is conducted. If MRS is available, preservation of the NAA/Cr ratio is assessed. Concurrently, the immunological status is evaluated by measuring pro-inflammatory cytokine levels [78]. As shown in research, the inflammatory microenvironment can lead to increased expression of MHC class II on MSCs and enhance their immunogenicity. Moreover, the presence of induced alloantibodies against a previous infusion is associated with reduced survival of repeatedly administered cells [199]. The decision to repeat an MSC course is based on the absence of clinically significant adverse effects, stable or improved clinical status according to SARA and mRS scales, absence of negative dynamics on MRI/MRS, and controlled levels of pro-inflammatory markers [200].

At 12 months, a full evaluation cycle is completed. Plasma NfL levels are reassessed to confirm stabilization of the neurodegenerative process [187], along with LSD-specific biomarkers (GAGs, GM2, sulfatides, galactosylceramide, etc.) to evaluate clearance of accumulated substrates, as well as cytokine dynamics; a reduction in cytokines confirms the immunomodulatory potential of MSCs [78]. If positive dynamics or stabilization is observed across all parameters (clinical scales, neuroimaging, biomarkers), repeated courses are planned. Before each subsequent administration, a complete blood count, neurological assessment, and VEGF level monitoring for safety control are performed. Thus, the proposed monitoring system allows for objective assessment of the effects of MSC therapy in LSDs and timely decision-making regarding treatment continuation or adjustment.

## 11. Cost, Accessibility, Logistics, and Alternatives

Despite the significant therapeutic potential of MSCs in CNS diseases, their translation into widespread clinical practice is accompanied by a number of economic, logistical, and technological limitations. Currently, most cell-based technologies are at the stage of preclinical studies or early-phase clinical trials, which limits their application in routine medical practice [201].

Cell therapy belongs to the category of high-tech medical care and requires a complex infrastructure for the production and quality control of cell products. The manufacture of cell-based preparations involves compliance with GMP standards, microbiological and genetic testing, as well as functional activity assessment of cells, which significantly increases the cost of therapy. Furthermore, cell products require individualized manufacturing, which limits scalability and raises treatment costs. As a result, cell therapy remains expensive and is available primarily within specialized clinical centers and research programs. High costs are also characteristic of other modern biomedical technologies, including gene therapy used for hereditary nervous system disorders and LSDs [202].

The availability of cell therapy is limited by both economic and regulatory factors. Despite the large number of experimental studies, most MSC-based cell products have not yet received widespread clinical application [203]. Nevertheless, clinical studies indicate that MSC administration is relatively safe and represents a potentially promising direction for the treatment of various neurological diseases, including CNS injuries and neurodegenerative disorders [204].

The logistics of cell therapy is a complex process involving the procurement of biological material, cell culture, cryopreservation, transportation, and preparation of the product for clinical use. Transport of cell products is most often carried out in a cryopreserved state at ultra-low temperatures. Violation of storage conditions can lead to reduced cell viability and diminished therapeutic efficacy.

Alternative therapeutic approaches include gene therapy, gene-cell therapy, and enzyme replacement therapy. Gene therapy is one of the most promising directions for the treatment of hereditary neurodegenerative diseases and LSDs. This approach is based on the delivery of a functional copy of the defective gene using viral vectors, most commonly AAV or lentiviruses [205]. Gene-cell therapy is a combined approach based on ex vivo genetic modification of autologous patient cells followed by their transplantation. The procedure involves harvesting a target cell population, genetically modifying them, and returning them to the patient’s body to achieve a therapeutic effect. Enzyme replacement therapy is the most extensively studied and widely used pathogenetic treatment method for LSDs. This approach is aimed not at correcting the genetic defect, but at compensating for its biochemical consequences through exogenous administration of the missing enzyme [206].

Currently, active research into new therapeutic strategies for CNS diseases continues. MSCs are considered a promising platform for the development of combined therapeutic approaches due to their immunomodulatory, neuroprotective, and regenerative properties [207]. Contemporary studies are also exploring the possibility of genetic modification of MSCs, the use of EVs, and the combination of cell therapy with gene therapy to enhance the efficacy of treatment for neurodegenerative diseases.

Crucially, a major regulatory caveat must also be acknowledged: most MSC- and MSC-EV-based approaches for CNS disorders and LSDs remain investigational, with no broad marketing authorization in most jurisdictions.

## 12. Current Challenges and Future Perspectives

Despite decades of intensive research, MSC-based therapies have not yet achieved the widespread clinical breakthrough long anticipated for neurodegenerative diseases and LSDs. Regulatory pathways for MSC products remain fragmented. The FDA regulates MSCs as biological products requiring IND and BLA, while EMA classifies them as ATMPs, yet neither agency provides disease-specific guidance for neurodegenerative disorders, and potency assay standardization remains a major unresolved hurdle. For MSC-derived EVs, the regulatory framework is even less defined, with open questions on classification, stability criteria, and suitable quality controls.

A fundamental limitation of MSC therapy is the poor survival and engraftment of transplanted cells. Following administration, MSCs encounter a hostile host microenvironment characterized by oxidative stress, inflammatory mediators, and hypoxia, all of which impair their survival and functional capacity [208]. The vast majority of intravenously administered MSCs are rapidly cleared from the circulation, with pulmonary entrapment accounting for the loss of up to 80–90% of infused cells. Even when cells reach target tissues, their persistence is typically transient, with most studies reporting detectable cells for only days to weeks after administration [209].

The discrepancy between controlled in vitro conditions and the challenging in vivo environment creates a fundamental barrier to cell engraftment. Factors such as donor age, culture conditions, and the pathological microenvironment all constrain MSC activity and functional plasticity, limiting the stability and reliability of clinical outcomes [210]. Moreover, MSCs in the diseased host may undergo apoptosis shortly after transplantation, and their therapeutic effects may instead be mediated through the release of paracrine factors or apoptotic vesicles [208].

Although MSCs are considered hypoimmunogenic due to low expression of MHC class II and co-stimulatory molecules, they are not immunologically inert. Cellular and humoral immune responses can develop against allogeneic MSCs, including the appearance of anti-donor antibodies, which may reduce the efficacy of repeated administrations. The clinical significance of this phenomenon requires further evaluation, but the potential for immune rejection poses a particular challenge for repeated dosing regimens that are likely necessary to maintain therapeutic benefit. MSC-derived extracellular vesicles, while retaining many therapeutic properties of their parent cells and offering improved safety profiles, may also elicit immune responses, particularly upon repeated administration [211]. Notably, both MSCs and EVs can inhibit immune cell proliferation, but they may induce different patterns of antibody responses, with implications for long-term use.

The optimal timing of MSC administration relative to disease stage remains poorly defined. In preclinical studies, MSCs are typically administered at or shortly after disease induction, often before significant pathology has developed. In clinical practice, patients are diagnosed at much later stages, after substantial neuronal loss has already occurred. This discrepancy in timing likely contributes to the limited efficacy observed in clinical trials, particularly in neurodegenerative diseases where the therapeutic window may have already closed [212]. Similarly, the question of single versus repeated dosing remains unresolved. Evidence from preclinical models suggests that repeated dosing may be necessary to achieve sustained immunomodulatory effects. This is consistent with observations from early-phase clinical trials, where single administrations have generally failed to demonstrate robust, durable efficacy. The duration of therapeutic effect is also unclear. Most clinical studies have reported relatively short-lived benefits, raising questions about whether the biological effects of MSCs, which are largely paracrine and transient, can truly modify the trajectory of chronic diseases.

The most rational path forward lies in combination strategies using MSCs as adjuncts to gene or enzyme replacement therapy and in engineered MSC platforms designed for sustained, targeted delivery.

## 13. Conclusions

The therapeutic promise of MSCs in CNS disorders rests not on neuronal replacement, but on their pleiotropic capacity to modulate neuroinflammation, support repair, and—in LSDs—enable enzymatic cross-correction. However, the field remains at an exploratory stage, and several constraints temper enthusiasm.

Translation is hindered by source-dependent variability in safety and efficacy (BM-MSCs offer superior hemocompatibility; adipose/perinatal sources show higher secretory activity but increased thromboembolic risk), unresolved dosing and administration routes, and the lack of standardized GMP-compliant protocols. MSC-EVs, while attractive as cell-free candidates with potential BBB interaction, face major hurdles in yield, batch-to-batch consistency, and regulatory qualification [20,46].

The strongest clinical evidence base to date exists for multiple sclerosis, amyotrophic lateral sclerosis, Parkinson’s disease, and Alzheimer’s disease, yet convincing, long-term efficacy in large, placebo-controlled trials remains elusive. In LSDs, the most encouraging data come from combination approaches with hematopoietic stem cell transplantation, but robust clinical validation is still pending [64,160].

Critical unknowns include durability of effects after repeated dosing, predictive biomarkers, optimal intervention timing, and whether genetically modified MSCs or EVs can achieve sustained enzymatic correction without immunogenicity or off-target effects [6,168].

Thus, MSC-based therapy for neurodegenerative diseases and LSDs is at the stage where moving forward requires large randomized placebo-controlled trials with harmonized endpoints, rigorous product standardization, and multimodal monitoring. The most rational path lies in combination strategies—MSCs as adjuncts to gene or enzyme replacement therapy—and engineered platforms for sustained targeted delivery. Crucially, all applications should remain within approved trial frameworks until regulatory approval is obtained, while unproven commercial offerings outside these settings should be unequivocally discouraged [5].

## Figures and Tables

**Table 1:** Comparison of MSC sources.

MSC Source	Advantages/Key Properties	Limitations/Risks	Safety and Hemocompatibility
Bone marrow	– Well-studied, extensive clinical experience [63] – High immunomodulatory activity [13] – Pronounced osteogenic potential [44] – Low TF expression [20]	– Invasiveness of harvest, limited aspirate volume – Decline in proliferative and differentiation potential with donor age [58] – Donor-dependent variability [53]	– Relatively safe for intravenous administration [20] – Minimal risk of thrombosis – No hypersensitivity reported [64]
Adipose tissue	– High cell yield from lipoaspiration [63] – Higher proliferation compared to BM-MSCs – Strong secretion of growth factors (bFGF, IGF-1) [44] – Pronounced immunosuppressive effect (↑HGF, ↑IL-10) [48]	– Invasiveness of the procedure (less traumatic than bone marrow harvest but still requires surgical intervention) [58]	– High TF expression, risk of thromboembolism upon intravenous administration; hemocompatibility assessment required [20]
Umbilical cord blood	– Non-invasive collection [63] – Young, “primitive” MSCs with low immunogenicity [65] – The secretome is rich in neurotrophins and survival factors [57]	– Low MSC frequency in the sample; isolation efficiency ≤ 60% [65] – Limited availability of cord blood banks – Variability in isolation success [58]	– Stable karyotype, non-tumorigenic [65]
Umbilical cord/placenta	– Available without risk to mother or child – Highest proliferative potential [48] – Rich secretory profile [57] – Minimal ethical and legal restrictions [65]	– Allogeneic only; infection screening and HLA typing required [53] – Heterogeneity of properties depending on harvest site and protocol [65] – High TF expression [20]	– Risk of coagulopathy and thromboembolism upon infusion [20,53] – Stable karyotype, non-tumorigenic

**Table 2:** Comparison of MSC administration routes.

Route of Administration	Invasiveness	CNS Coverage	Advantages	Disadvantages
Intravenous	Minimal [70]	Systemic distribution. Indirect and generally limited for brain parenchyma. A significant portion of MSCs is initially retained in the lungs, limiting cell access to the brain [89].	– Technically the simplest route of administration; Does not require neurosurgical intervention [66]; – Convenient for repeated administrations.	– Most cells do not reach the brain [67]; – Requires high hemocompatibility [20]; – Effect on the CNS is mediated, not direct; – AEs upon intravascular administration may include thromboembolic complications associated with highly procoagulant MSC products [53].
Intrathecal	Moderate—requires lumbar puncture but does not require intracranial or stereotactic access [90].	Distribution via CSF [91]; broader than local parenchymal injection, but without evidence of uniform coverage of all CNS regions [90].	– Bypasses the BBB [89]; – Potentially provides wider CNS access than local focal routes [92]; – Studies in neurological diseases have shown a generally acceptable safety profile [93].	– Requires lumbar puncture; – Uneven distribution: upper brain regions receive fewer cells; – Procedural adverse events possible, with a slight increase in non-serious musculoskeletal and connective tissue events noted [93]; – CSF entry does not guarantee effective homing to the target area, and migration efficiency remains a limitation [90]; – CSF may alter the transcriptome and secretory profile of MSCs [94].
Intraventricular	High—requires intracranial access; repeated administration typically uses an Ommaya reservoir [81].	Wide coverage of CSF spaces, distribution broader than local parenchymal injection, and may include the brain and cervical spinal cord, but uniform CNS coverage has not been demonstrated [81].	– Direct delivery of cells to the ventricular system and CSF, bypassing the BBB; – Permanent port allows multiple administrations without repeated punctures [93]; – Provides greater brain cell localization than the IV route in preclinical models [95].	– Requires intracranial access and catheter/reservoir placement; – Procedural adverse events possible, including reservoir site pain and headache. Fever, headache, nausea, and vomiting may occur after administration [81].
Intraparenchymal (local brain)	Very high—requires stereotactic neurosurgery and direct access to brain tissue [96].	Localized, restricted to the implantation area; suitable for targeted delivery to a selected anatomical region but not for broad CNS coverage.	– Provides maximally targeted delivery to the selected brain region; – Bypasses the BBB; – Clinical feasibility demonstrated in early human studies [97].	– Requires stereotactic neurosurgery [96]; – Risk of vascular damage and hemorrhage [98]; – Potential technical delivery issues, including cannula occlusion and heterogeneous cell distribution [98].

**Table 3:** Summary of clinical and translational evidence for MSC-based therapies in neurological and lysosomal storage diseases.

Disease	Evidence Level	MSC Source	Route	Main Outcome	Key Limitation
Multiple sclerosis	Phase I/II, open-label	Autologous BM-MSCs	IV	Treatment was feasible and generally well tolerated; possible neuroprotective effects were observed	Small, uncontrolled study; efficacy could not be confirmed in a controlled setting
	Phase II, controlled	Autologous BM-MSCs	IV	Favorable safety profile; modest effects on MRI inflammatory activity	Primary efficacy endpoint was not met; convincing disease-modifying efficacy has not been established
	Phase I/II, randomized double-blind crossover (MESEMS)	Autologous MSCs	IV	Designed to assess safety and efficacy in active MS	Represents higher-level evidence than uncontrolled studies; should be interpreted separately from early open-label studies
	Open-label Phase II	Autologous MSC-NPs	IT	Changes in CSF biomarkers and some clinical improvement were reported	No placebo control, limiting interpretation of efficacy
Amyotrophic lateral sclerosis	Phase I	Autologous BM-MSCs	IT	Acceptable tolerability; study primarily established safety	Not powered to demonstrate clinical efficacy
	Phase II	Autologous BM-MSCs	IT	Short-term changes in ALSFRS-R and CSF biomarkers were observed	No sustained benefit on long-term outcomes/survival; effects appeared transient
	Phase III, randomized double-blind placebo-controlled	Autologous BM-MSC-NTF (NurOwn)	IT	Biological activity and biomarker changes were observed	Primary efficacy endpoint was not met; clinical efficacy was not confirmed in the overall population; the trial included 196 randomized participants and is listed as completed in ClinicalTrials.gov
Alzheimer’s disease	Phase I	Allogeneic UCB-MSCs	ICV	Feasible and generally tolerable; transient fever and CSF inflammatory changes occurred	Acute inflammatory reactions; small early-phase study
	Phase I/IIa, randomized placebo-controlled	Allogeneic UCB-MSCs	ICV	Biomarker changes were observed	No significant clinical benefit; more fever, headache, nausea and vomiting in MSC group
	Phase IIa	UCB-MSCs ± dexamethasone	ICV	Investigated safety and exploratory efficacy	Clinical benefit remained unproven; inflammatory reactions required consideration of dexamethasone
Parkinson’s disease	Early-phase, uncontrolled	Autologous/allogeneic BM-MSCs	Intraparenchymal	Feasibility and acceptable safety; some improvement in UPDRS reported	Small uncontrolled studies; limited evidence of efficacy
Spinocerebellar ataxia	Early clinical study	Allogeneic UC-MSCs	IT	Improvements in ICARS and ADL scores were reported	Small uncontrolled evidence; durability and causal efficacy remain uncertain
Metachromatic leukodystrophy	Clinical case/limited clinical series	MSCs; source reported as expanded MSCs	IV/combined with HSCT	Some patients showed improved nerve conduction; one case showed neurological stabilization and normalization of ARSA activity	Very small numbers; absence of controlled comparison prevents attribution of benefit specifically to MSCs
Cerebral adrenoleukodystrophy	Clinical case series (*n* = 2)	Allogeneic MSCs	IT	Administration was feasible and uncomplicated	Demyelination continued despite treatment, providing an important negative clinical observation
Krabbe disease	Preclinical animal evidence	BM-MSCs/AD-MSCs	ICV/intracerebral	Reduced CNS inflammation and slowed myelin loss in mouse models	No established clinical efficacy; evidence remains preclinical
Tay–Sachs/Sandhoff disease	Preclinical animal evidence	Genetically modified MSCs/MSC combinations	IV/transplantation	Enzyme delivery and partial metabolic correction demonstrated in animal models	No direct clinical application of MSCs has been established

## Data Availability

No new data were created or analyzed in this study. Data sharing is not applicable to this article.
